# Industrialized human gut microbiota increases CD8+ T cells and mucus thickness in humanized mouse gut

**DOI:** 10.1080/19490976.2023.2266627

**Published:** 2023-10-18

**Authors:** Pajau Vangay, Tonya Ward, Sarah Lucas, Lalit K. Beura, Dominique Sabas, Max Abramson, Lisa Till, Susan L. Hoops, Purna Kashyap, Ryan C. Hunter, David Masopust, Dan Knights

**Affiliations:** a Bioinformatics and Computational Biology Program, University of Minnesota, Minneapolis, MN, USA; b BioTechnology Institute, University of Minnesota, Minneapolis, MN, USA; c Department of Biology, Syracuse University, Syracuse, NY, USA; d Department of Molecular Microbiology and Immunology, Brown University, Providence, RI, USA; e Department of Food Science and Nutrition, University of Minnesota, Minneapolis, MN, USA; f Department of Neuroscience, Macalester College, St. Paul, MN, USA; g Department of Neuroscience, Albert Einstein College of Medicine, Bronx, NY, USA; h Division of Gastroenterology and Hepatology, Department of Internal Medicine, Mayo Clinic, Rochester, MN, USA; i Department of Computer Science and Engineering, University of Minnesota, Minneapolis, MN, USA

**Keywords:** Microbiome, microbiota, immigration, inflammation, mucosal barrier, infection, diet

## Abstract

Immigration to a highly industrialized nation has been associated with metabolic disease and simultaneous shifts in microbiota composition, but the underlying mechanisms are challenging to test in human studies. Here, we conducted a pilot study to assess the differential effects of human gut microbiota collected from the United States (US) and rural Thailand on the murine gut mucosa and immune system. Colonization of germ-free mice with microbiota from US individuals resulted in an increased accumulation of innate-like CD8 T cells in the small intestine lamina propria and intra-epithelial compartments when compared to colonization with microbiota from Thai individuals. Both TCRγδ and CD8αα T cells showed a marked increase in mice receiving Western microbiota and, interestingly, this phenotype was also associated with an increase in intestinal mucus thickness. Serendipitously, an accidentally infected group of mice corroborated this association between elevated inflammatory response and increased mucus thickness. These results suggest that Western-associated human gut microbes contribute to a pro-inflammatory immune response.

## Background

We previously characterized gut microbial changes in humans migrating from a recently industrialized nation, Thailand, to a highly industrialized nation, the US, with the aim to understand the high and increasing prevalence of metabolic diseases in modern industrialized nations. We observed that moving to the US is associated with a profound loss of microbial diversity, systematic loss of native bacterial taxa, and the displacement of the non-Western-associated *Prevotella* genus with Western-associated *Bacteroides* genus.^1^ Although we found that loss of microbiome diversity was strongly associated with obesity in that study, we were not able to make conclusions about causality because the study was observational. One clear hypothesis coming out of that study was that human gut microbiota changes induced by industrialized lifestyle and diet are associated with detrimental effects on gut health and overall metabolic health. Detrimental effects of industrialized lifestyle and diet on human health may be mediated by concomitant changes in gut microbiota. For example, another recent study provided evidence that a low-fiber diet typical of industrialized society can cause extinction of certain taxa and depletion of the protective mucus lining along the gut.^2^

The purpose of this study was to follow up on our previous human study by testing for causal effects in a well-controlled animal model. Here, we conducted a pilot study using a humanized germ-free mouse model to test whether there are detrimental effects of a low-fiber diet, indicative of a Western diet, on metabolic health and whether these effects are dependent on having a westernized microbiota. We also profiled intestinal lymphocyte populations and measured colonic mucus thickness to test whether changes in inflammation and gut mucosal barrier are associated with a westernized diet, a westernized microbiota, or both.

## Results

### US-donor microbiota induces alterations in intestinal T cell composition, indicative of an inflammatory immune response

**Figure 1.**
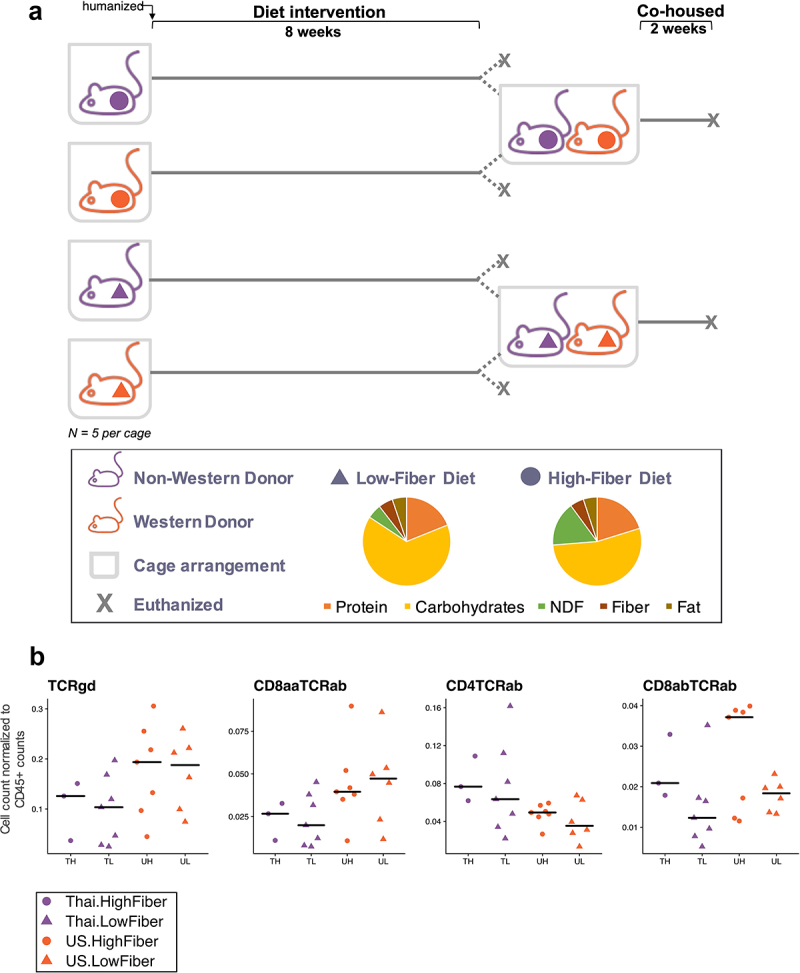
Low-fiber diet induces deleterious metabolic responses in humanized mice (1a) mouse experimental study design included mice that received donor microbiota from Thai (T) or US (U) human donors, and were placed on either a high-fiber (H) or low-fiber (L) diet for 8 weeks, resulting in four experimental groups: TH = Thailand-donor-high-fiber; UH = US-donor-high-fiber TL = Thailand-donor-low-fiber; UL = US-Donor-low-fiber. After an 8-week diet intervention, *n* = 2 mice in each group were euthanized. The remaining mice were cohoused by diet: TH and UH were cohoused, and TL and UL were cohoused. Cohousing was performed for only donor pair 1. NDF = non-digestible fiber. (1b) TCRγδ+ and CD8ααTCRαβ+ T cells are significantly elevated in US-donor groups (two-way ANOVA, donor *P* = .035 and *P* = .027, respectively), while CD4TCRαβ+ T cells is significantly elevated in Thailand-donor groups (two-way ANOVA, donor *P* = .016). CD8αβTCRαβ+ T cells are elevated in groups consuming a high fiber (two-way ANOVA, diet *P* = .032). All other p-values were not significant (>.05). All measurements were taken at week 8 and exclude *n* = 4 infected mice from the TH group. Points represent counts of live immune cell types normalized by the total count of live CD45+ per mouse small intestine.

We performed fecal microbiota transplantation into germ-free mice using previously collected human stool^1^. Human stool was collected from four individuals with gut microbial distances closest to the centroid of their respective Thailand or US groups, and each Thailand and US donor was matched on age, ethnicity, sex, and BMI, resulting in two donor pairs. We gavaged 4–7-week-old germ-free C57BL/6 mice (*n* = 38) with individually prepared human stool samples. After humanization, the mice were fed either a high-fiber (H) or low-fiber (L) diet. As a result, the mice were grouped into eight donor-diet groups (*n* = 4–5 mice per group) and placed in individually ventilated cages (Figure 1a). Although donor microbiota samples were not pooled prior to gavage, the work presented here includes analyses of microbiomes by diet and individual donor or by diet and donor type (TH=Thailand-Donor-High-Fiber; UH=US-donor-High-Fiber TL=Thailand-Donor-Low-Fiber; UL=US-Donor-Low-Fiber). Mice were sacrificed after 8 weeks on their respective diets. To understand the dominance of Thailand or US gut bacterial taxa in a shared environment, a subset of mice that received donor material from donor-pair-1 were co-housed within their diet groups for an additional 2 weeks (TH with UH and TL with UL) (Figure 1a).

Using flow cytometry to characterize immune cell populations in the gut intra-epithelial compartments, we found that after 8 weeks, enumeration of intraepithelial lymphocytes revealed a primarily microbiota-dependent response rather than a diet-dependent response. We found an elevated percentage of TCRγδ+ and CD8ααTCRαβ+ T cells in US-donor groups (*P* = .035 and *P* = .027, respectively) and observed a reduced frequency of CD4TCRαβ+ T cells in US-donor groups (*P* = .016, Figure 1b), independent of diet. Interestingly, we found that the high-fiber diet induced an elevation in CD8αβTCRαβ+ T cells (*P* = .032), which was independent from and not synergistic with the inflammatory contributions of the US-donor microbiota. Examination of immune cell populations in the lamina propria revealed similar trends (Supplemental Figure S1).

### US and Thailand donor microbiota yield dissimilar gut epithelial morphologies

**Figure 2.**
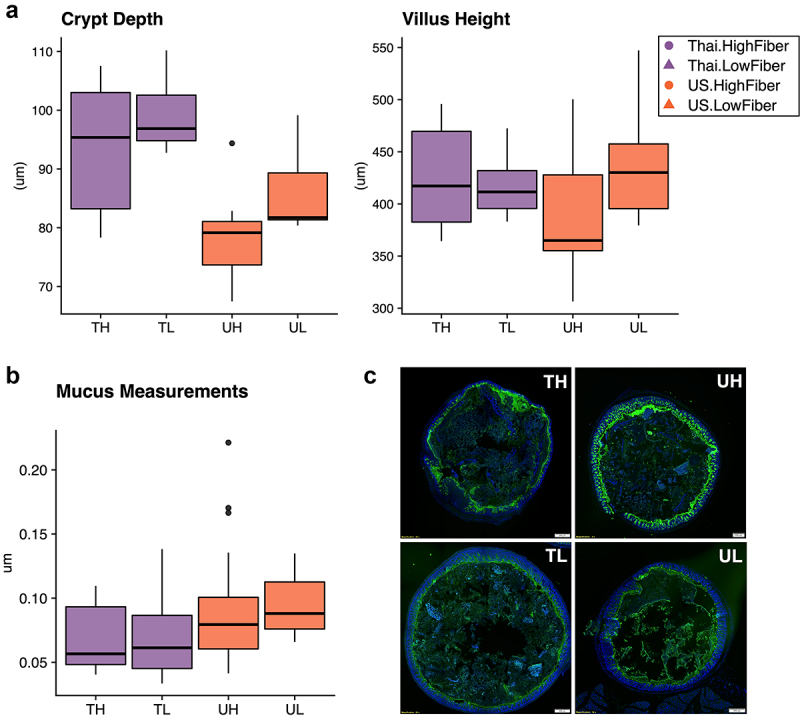
Immune responses in the ileal intra-epithelial compartments, intestinal villi height, and colonic mucus thickness vary by donor microbiota (2a) duodenal crypt depth is significantly deeper in Thailand-donor groups than US-donor groups (two-way ANOVA, donor *P* = .00034 and diet *P* = .062). Villus height measurements were not significantly different by donor or diet types. (2b) colonic mucus measurements (three measurements taken per cross-sectional image, measured in triplicate and normalized by researcher) were thicker in Thailand-donor groups (two-way ANOVA, donor *P* = .0048). (2c) representative images of samples from TH, TL, UH, and UL groups show DAPI stains of cross-sectional colonic sections used for mucus measurements (TH = M13 from Thailand-donor-high-fiber group; TL = M22 from Thailand-donor-low-fiber group; UH = M31 from US-Donor-high-fiber group; UL = M7 from US-Donor-low-fiber group).

At 8 weeks, we measured the villus height and crypt depth of the gut epithelium in the duodenum, and found that while there were no significant differences in villus height across donors and diets, Thailand-donor groups (TL and TH) had significantly deeper crypts (*P* = .00034) (Figure 2a). At this same time point, we also measured mucin thickness in the colon and found that on average, US-donor groups exhibited thicker layers of mucus compared to Thailand-donor groups (*P* = .0048). Contrary to our hypothesis that a Thailand gut microbiota and a high fiber diet together would contribute to increased colon mucin thickness, we found that the US-Donor-Low-Fiber (UL) group exhibited a median mucin thickness that was more than 1.5 times that of the Thailand-Donor-High-Fiber (TH) group Figure 2(b,c). The thicker mucus layer combined with the pro-inflammatory markers found in the US-donor groups led us to consider an alternate explanation that a pro-inflammatory US microbiota was also inducing mucin production via an infection-like pro-inflammatory response.

### Infection in mice increases mucin production and inflammatory markers

**Figure 3.**
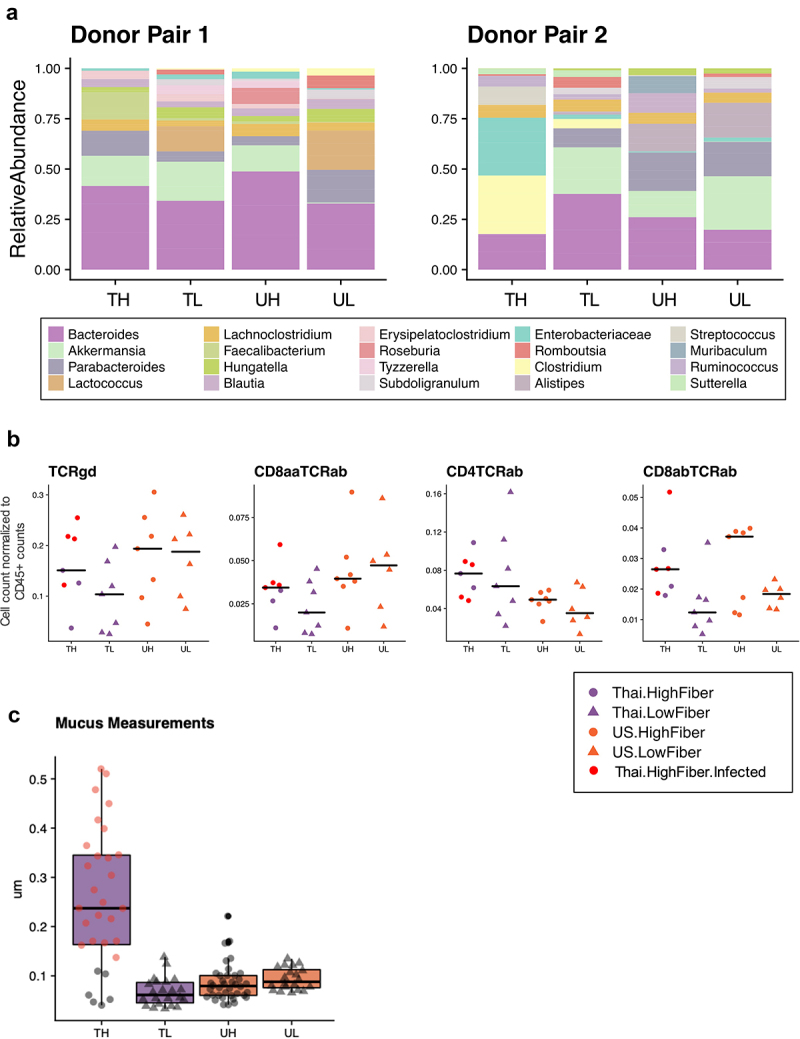
Infection in mice increases mucin production and inflammatory markers (3a) the relative abundances of the 15 most prevalent taxa within each donor pair are shown. Samples were collected at study endpoints (week 8 or 10, depending on whether or not the mouse was cohoused). Note the bloom of *Enterobacteriaceae* (FDR adjusted q-value = 6.22e-5) and *Clostridium* (FDR adjusted q-value = .071) in the TH group of donor pair 2, which are composed entirely of the infected mice. Other proteobacteria, enterobacterales, was also elevated in the infected mice (FDR adjusted q-value = .013), but was not prevalent enough in all groups to be shown. TH = Thailand-donor-high-fiber; UH = US-donor-high-fiber; CH = Cohoused TH and UH; TL = Thailand-donor-low-fiber; UL=US-Donor-low-fiber; CL = Cohoused TL and UL. (3b) inclusion of the infected mice elevates both TCRγδ+ and CD8ααTCRαβ+ T cells in the TH group such that the differences between donor groups are no longer significant (two-way ANOVA, donor *P* = 0.147 and *P* = .055, respectively). Furthermore, inclusion of the infected mice in the TH group also elevates the counts of CD4TCRαβ+ and CD8αβTCRαβ+ T cells, such that their previous associations are more pronounced (one-way ANOVA, donor *P* = .012 and diet *P* = .0079, respectively). Points represent counts of live immune cell types normalized by the total count of live CD45+ cells per mouse small intestine at week 8, and include the *n* = 4 infected mice in the TH group. (3c) infection in mice leads to increased colonic mucus thickness in the TH group compared to all other groups (one-way ANOVA, *P* = 6e-16). Points represent mean colonic mucus measurements (five measurements taken per cross-sectional image, measured in triplicate and normalized by researcher). Points in red indicate measurements from the *n* = 4 infected mice.

This study included a serendipitous finding in a subset of mice in the Thailand-High-Fiber (TH) group that acquired an unexplained and unintended infection following their gavage with the Thailand-donor microbiota (Table S1). Visual inspection showed that these mice exhibited enlarged spleens, abnormally white cecums, unusual nodule-like gut adipose tissue, and pale-colored livers. A comparison of two groups of mice gavaged from the same donor material, although placed on different diets, show drastic taxonomic differences over 8 weeks (Supplemental Figure S2a). Of note, taxa found to be significantly elevated in the infected mice include potentially pathogenic taxa, such as *Clostridium*, *Enterobacteriaceae*, and Enterobacterales (Figure 3a). Interestingly, *Akkermansia muciniphila* was undetectable in the group of infected mice, despite it being present in every other group of mice, regardless of donor (Figure 3a).

In our previous comparisons, the levels of TCRγδ+ and CD8ααTCRαβ+ T cells were significantly lower in Thailand-donor groups compared to US-donor groups (Figure 1b). Infected mice, despite having received Thailand-donor microbiota, exhibited high levels of these two pro-inflammatory immune cell types, such that comparisons between donor groups when including these mice were no longer significantly different (TCRγδ+ *P* = .056, CD8ααTCRαβ+ T cells *P* = .28) (Figure 3b). Because CD4TCRαβ+ T cells were already elevated in Thailand-donor groups previously, the addition of the infected mice resulted in little change between group comparisons (without infected mice *P* = .016, and with infected mice *P* = .012). Furthermore, infected mice exhibited colonic mucus that was as high as five times thicker than those of non-infected mice (Figure 3c). This finding provided corroborating evidence for the theory posited above that the increased mucus thickness observed in the US-donor mice was concomitant with an infection-like pro-inflammatory response. While the absence of mucin-degrading *A. muciniphila* may have contributed to the thicker mucus layer in the infected mice, we did not find a correlation between the relative abundance of *A. muciniphila* and mucus thickness measurements in other samples (Figure S2B).

### A low-fiber diet leads to deleterious metabolic outcomes independent of microbiota source

**Figure 4.**
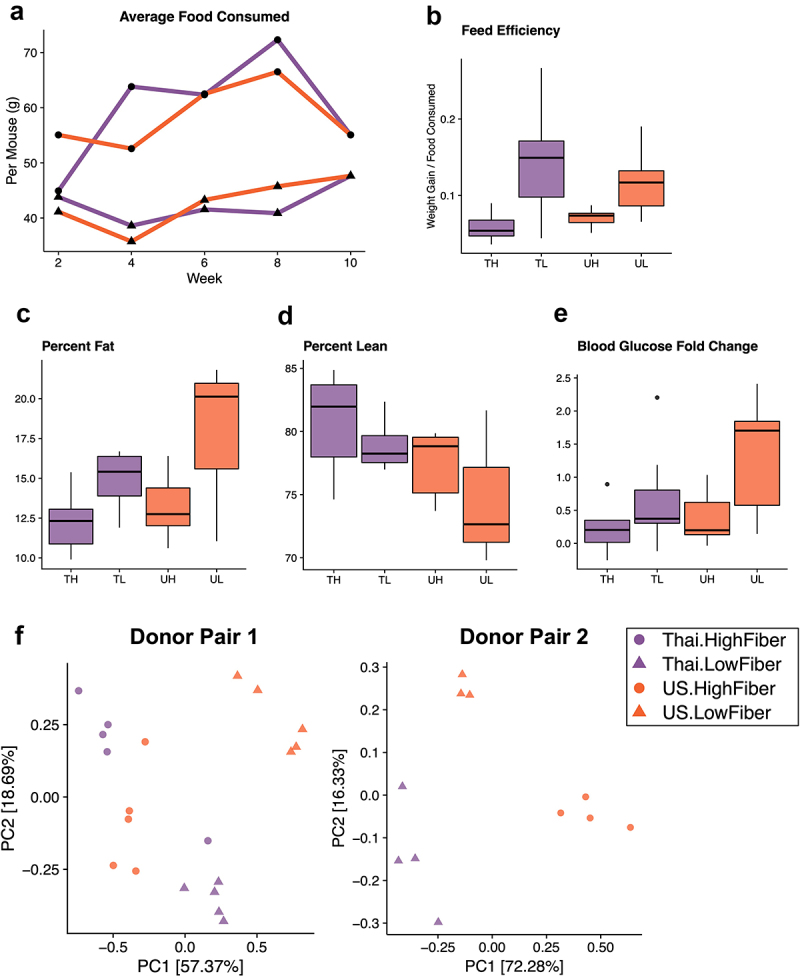
A low-fiber diet leads to deleterious metabolic outcomes (4a) average food consumption per mouse (chow was weighed for each cage at two-week intervals and averaged based on the number of mice per cage) throughout the study period revealed that low-fiber diet groups consistently consumed less food than high-fiber diet groups. (4b) groups consuming a low-fiber diet are more efficient at converting their feed into body weight gain (two-way ANOVA, diet *P* = 3.2e-05). Feed efficiency is calculated as the gain in body weight per mouse by the average grams of chow consumed per mouse between weeks 6 and 8. (4c) groups consuming a low-fiber diet gain more body fat than those on a high-fiber diet (two-way ANOVA, diet *P* = .013). Body fat was measured at the end of the 8-week diet intervention. (4d) Thai-donor groups gained more lean mass than US-donor groups (two-way ANOVA donor *P* = .032). (4e) groups consuming a low-fiber diet exhibited a significant difference in fasting blood glucose fold change (two-way ANOVA, diet type variable *P* = .012). Fasting blood glucose measurements were taken at baseline (prior to gavage) and at the end of the 8-week diet intervention (time of sacrifice). (4F) PCoA of mouse microbiota (weighted UniFrac) at 8 weeks show clustering by diet (ANOSIM *R* = .7267, *P* = .001) and donor microbiota (ANOSIM *R* = .09222, *P* = .119) in donor pair 1, and by diet (ANOSIM *R* = .9656 and *P* = .004) and donor microbiota (ANOSIM *R* = .5767 and *P* = .01) in donor pair 2. PCoAs are separated by donor-pair for clarity and to observe the differences in donor pair gut microbiota. Due to the outlier effects of the infected mouse samples, donor pair 2 Thai-high-fiber samples are excluded. TH = Thailand-donor-high-fiber; TL = Thailand-donor-low-fiber; UH = US-donor-high-fiber; UL = US-Donor-low-fiber. See also Figure S3 and Table S2.

We found that regardless of donor microbiota, consumption of a low-fiber diet induced adverse metabolic outcomes. Although low-fiber diet groups (TL and UL) consistently consumed less food throughout the study by weight (Figure 4a), this behavior was likely due to the extraction of more energy from the low-fiber diet, which had sucrose as its primary carbohydrate source (*P* = 3.2e-05) (Figure 4b). As a result, at the end of 8 weeks, the low fiber diet groups (TL and UL) exhibited significantly higher percent body fat than the high fiber diet groups (TH and UH) (*P* = .013) (Figure 4c). Despite the diet-induced increase in body fat, Thailand-donor groups developed more lean mass than US-donor groups (*P* = .032) (Figure 4d). Compared to the high-fiber diet groups, both low-fiber diet groups also had a significantly elevated blood glucose response when compared against their baseline blood glucose response (at the time of humanization) (*P* = .012) (Figure 4e).

Analysis of fecal pellets collected at 8 weeks showed that while microbial composition is influenced by donor microbiota, the majority of taxonomic differences are driven by diet (Figure 4f, Figure S3). Comparison of the four groups (TH, TL, UH, UL) showed that *Butyricicoccus*, *Dorea*, and *Gemmiger* were more abundant in the high-fiber diet groups, and *Subdoligranulum, Romboutsia, Lactococcus, Enterococcus, Flavonifractor, Terrisporobacter* were more abundant in the low-fiber diet groups (q < .10, Figure 3a, Figure S3). Although 15 of the 18 significantly differentiated taxa (q < .10) are associated with diet, several taxa, *Muricomes*, *Negativibacillus*, and *Collinsella*, were associated with the donor type and found to be more abundant in US-donor groups (Figure S3, Table S2). Beta diversity analysis showed a lack of consistent clustering by diet or donor in the overall microbiome profile prior to cohousing (Figure S6). Cohousing mice for an additional 2 weeks (Donor Pair 1 only) resulted in compositional changes that also differed dependent on diet (Figure S4A). Cohousing Thailand- and US-donor mice on a low-fiber diet (TL and UL) resulted in an intermediary microbiota composed of bacterial taxa from both originating groups, but with more shared taxa with the US group (Figure S4B). Yet, cohousing Thailand- and US-donor mice on a high-fiber diet (TH and UH) promoted dominance of a Thailand-donor microbiota, which is primarily characterized by higher relative abundances of *Paraprevotella* and *Faecalibacterium*, members known for dietary fiber degradation. These findings suggest that proliferation of these commonly non-Western-associated bacterial taxa may be dependent on the consumption of non-digestible fibers.

## Discussion

In our pilot study with humanized germ-free mice, we found that a US-associated microbiota stimulated increased inflammatory response. This includes elevated TCRγδ+ T cells among intraepithelial lymphocytes in mice receiving US-donor microbiota. TCRγδ+ T cells have pronounced cytotoxic properties and produce inflammatory cytokines upon antigen recognition^3^, and a number of TCRγδ+ T cells have been elevated in models of colitis, environmental enteropathy, and celiac disease^4–6^. In contrast to the cytotoxic CD8 T cells, we observed a significant decrease in CD4 T cells in mice that received US microbiota. CD4 T cells in the gut are primarily composed of FoxP3+ regulatory T cells and anti-pathogenic Th1 and Th17 subsets^7^. This loss of a critical regulatory CD4 subset might further potentiate the inflammatory phenotype. Similar to the trend seen in the case of TCRγδ+ T cells, we also observed an increase in CD8ααTCRαβ+ T cells in US-donor microbiota groups. Compared to TCRγδ+ T cells, these T cells are considered immunoregulatory and shown to be protective against colitis^8,9^; however, their frequency is several orders of magnitude lower than the proinflammatory TCRγδ+ T cell, suggesting a predominantly inflammatory intestinal environment. CD8ααTCRαβ development is complex and has long been under debate. It exhibits T-reg-like behavior, requiring strong self-antigen interaction for positive selection^10^, which can also be disrupted by several cytokines upon entering the gut^11,12^. High-fat diets have been shown previously to cause low-grade inflammation^13,14^. Our study found that a high-fiber diet was associated with an expansion of CD8αβTCRαβ, induced intraepithelial lymphocytes (IELs) with previous exposure to microbial or dietary antigens and primarily cytolytic properties^15^. Despite their pro-inflammatory nature, these IELs can also take on protective roles that are critical for maintaining and restoring the integrity of the gut epithelium^16^.

Our pilot study also showed that the Thailand-donor microbiota were associated with significantly deeper crypts, independent of dietary fiber content. Previous work has shown that gut microbiota is important for the structural development of the gut epithelium, with germ-free mice exhibiting crypts that are less deep and villi that are thinner and longer than conventional mice^17^. High-fat diets have been shown to induce changes in villi morphology, primarily showing increased length for higher absorption of fats^18,19^, but limited changes in crypt depth^20^. Shallower crypt depth has been associated with chronic inflammation^21^.

One limitation of our pilot study is that biological measurements were taken from different tissue types within subjects. For example, the ileum was collected for immune cell profiling, and the duodenum was collected for crypt and villi measurements. Thus, our reported correlations may not be directly linked due to potentially biogeographical variability in the collected tissue sites, and future work controlling for this factor will be important for fully understanding the underlying mechanisms. Another limitation of this study is that we were unable to confirm the identity of the infectious agent in the infected mice nor the source of the infection. One possible hypothesis is that the combination of the specific donor material and diet created a gut microbial environment that enabled a preexisting pathogen to bloom; future experiments replicating this work will be important for validation.

## Conclusions

Despite the small sample size of our donors, our findings provide evidence that the westernized human gut microbiota may play a role in the promotion of inflammation in the gut. Interestingly, this pro-inflammatory response was associated with increased mucus thickness, a finding that was corroborated serendipitously by analysis of a group of accidentally infected mice. This supports the perspective that increased mucin production can sometimes be associated with infection^22–26^ and increased mucus thickness on its own may not be sufficiently indicative of a healthy gut environment. Important next steps for this study include validation studies with controlled infection models to fully understand the underlying mechanisms linking mucin production, inflammation, and infection. Future work should also take into account the immune profiles of the animals prior to gavaging, which is important to understand baseline immunity and account for relative changes in immunological responses over time. Altogether, our pilot study provides evidence of pro-inflammatory effects of westernized gut microbiota independent of dietary fiber consumption and suggests that these effects warrant further investigation.

## Methods

### Human study design and setting

Our previous human study included Hmong or Karen healthy females who were residing in the metropolitan Minneapolis-St. Paul area or in Thailand (a rural village in northwestern Thailand and a refugee camp in western Thailand). We collected stool samples from participants, conducted 24-h dietary recalls, measured BMI, and collected other relevant factors. Findings from this study can be found here^1^, and recommendations from our community-based participatory research approach can be found here^27^.

Of the human study participants, a subset of participants were invited to provide additional stool samples for follow-on animal studies based on their microbiome distance (beta diversity) to the centroid of their sample groups. In this mouse experiment, we used stool samples from four donors from the human study: two from the US and two from Thailand.^1^

### Microbiome sequencing and analysis

Described previously here.

### Mouse experimental design

Animal experiments were conducted at the Mayo Clinic and were approved by the Mayo Clinic Institutional Animal Care and Use Committee. Histology, mucus thickness measurements, and cell staining, isolation and cytometry procedures were performed at the University of Minnesota (UMN). Using a previous study^28^ on humanized mice with obese and lean phenotypes, in order to detect a significant difference in biodiversity between lean and obese mice with a significance level of 5%, power of 80%, and an effect size of 3.6, a power analysis yielded the minimum sample size per group to be 3 (exact calculation was 2.61). We chose to use 5 technical replicates per group to compensate for any potential failure of the gavage procedure and to allow for at least 1 mouse per group to be euthanized for morphological characterization at the end of 8 weeks.

### Mouse experiment specimen and data collection

We thawed 2 mL of previously collected frozen human stool over ice, then added it to a 15 ml conical tube containing 3 ml of pre-reduced PBS inside of a COY anaerobic chamber and vortexed for 1 min. 4–8-week-old germ-free C57BL/6 female mice were fasted overnight, then removed from germ-free isolators and gavaged with 300 uL of prepared donor material. Fasting blood glucose measurements were taken with a cheek bleed using a StatStrip Xpress glucometer, and mice were ear punched for identification. Mice were subsequently placed in cages with Sani-chips™and Crink-l’Nest™ bedding, which have been autoclaved at 128°C for 30 min with a 15-min dry cycle, and with nanopure drinking water which had been autoclaved in 1 L pyrex bottles at 121°C for 2 h. Prior to experiment start, mice were fed a standard autoclaved chow (LabDiet 5K67), and immediately after humanization, mice were placed on irradiated LabDiet 5061 or Harlan TD.86489. Cages were sealed and placed into an Arrowmight Maxi Seal IVC System, housed within a germ-free facility. Cages were changed, mice and chow were weighed, and pellets were collected every 2 weeks using sterile handling methods throughout the duration of the study. A mouse body composition analysis was performed for M11-M38 at study endpoints as previously described^29^. All mice were fasted overnight, then euthanized using CO_2_ asphyxiation. Heart blood was collected immediately post-euthanasia for fasting blood glucose tests. Gastrointestinal tracts were quickly removed, and colons were gently separated by cutting the cecum-colon junction and rectum.

### Histology

The duodenum was collected into 10 mL 10% formalin and sent to the UMN Comparative Pathology Shared Resource, where after 24 h, the tissue was transferred to 70% ethanol. Four-micrometer formalin-fixed, paraffin-embedded sections of tissue were deparaffinized and rehydrated, followed by hematoxylin and eosin staining. Images were taken using a NIKON Eclipse E 800 M microscope with 10× objective, and measurements were made using the NIS Basic Program.

### Thickness measurements of colonic mucus layer

Carnoy’s tissue fixation was employed as described previously^2,30^. Briefly, colon tissue was immediately incubated in fresh Carnoy’s fixative (dry methanol:chloroform:glacial acetic acid in the ratio 60:30:10) for 3 h. Tissue was transferred to fresh fixative for an additional 3 h. Colons were then washed in dry methanol for another 2 h, placed in cassettes, and stored in fresh dry methanol at 4°C. Samples were embedded in Paraplast paraffin embedding medium (Sigma Aldrich), and thin sections (~5–7 µm) were cut using a microtome (Leica RM2125 RTS) and deposited onto Superfrost Plus glass slides (Fisherbrand).

Sections were stained with 10 µg/mL *Ulex europaeus* agglutinin I (UEA-1) FITC conjugate (Vector Laboratories, Inc., Burlingame, CA) in 1X PBS for 30 min at 37°C^31^. Slides were incubated 2 × 3 min in wash buffer (215 mM NaCl, 20 mM Tris pH 7.5, 5 mM EDTA), then stained with 1 µg/mL 4’,6-diamidino-2-phenylindole (DAPI) in 1X PBS for 15 min at room temperature. Slides were again washed 2 × 3 min in wash buffer, then dropped for 3 min each in 50%, 80%, and 96% (v/v) ethanol and then air-dried. Coverslips were mounted on slides with Vectashield antifade mounting medium (Vector Laboratories, Inc.) and stored in a dark environment at 4°C. Images were acquired using an Olympus I×83microscope with a transmitted Koehler illuminator and a 4X objective lens using cellSens software (v.1.14, Olympus). Images were captured on a Hamamatsu ORCA-Flash4.0 V2 digital CMOS camera.

The images captured all of the available fecal masses of all mice, although this number was variable. Image sample names were blinded by S.L. and mucus thickness of the colonic sections was then measured in triplicate by P.V., D.S., and M.A. using ImageJ implemented in Fiji^32^. 1–2 images per fecal mass were taken per colon, 5 measurements per image were taken. Final measurements were normalized (mean) to account for differences between researchers, then averaged across three researchers.

### Cell isolation, staining, and flow cytometry

A two-inch section of the ileum was collected in 3% PFA, and the remainder of the small intestine was emptied of its contents and stored in 35 mL of CMF media. Tubes were stored in ice during transport. Leukocyte isolation from the small intestine was performed as previously described^33^. Briefly, for isolation of intraepithelial lymphocytes (IELs) from the small intestine, fecal contents were removed, Peyer’s patches were excised, and the gut was cut longitudinally and then into 1 cm pieces. Intestine pieces were incubated in 10% 1X HBSS/HEPES bicarbonate containing 15.4 mg/100 ml dithioerythritol (30 min at 37°C, 450 rpm) to extract IEL. After separating IELs, gut pieces were treated further with 100 U/ml type I collagenase (Worthington Biochemical, Lakewood, NJ, USA) for lamina propria lymphocyte (LPL) isolation. Single cell suspensions were surface-stained with antibodies to detect various leukocyte populations in the gut. The stained samples were acquired using LSR Fortessa flow cytometer (BD) and analyzed with FlowJo software (Treestar). A representative gating strategy is included in the supplemental materials (Figure S7).

## Supplementary Material

Supplemental Material

## Data Availability

The raw sequencing data for this study can be found in ENA with accession number PRJEB43065. Code to reproduce mouse microbiome analysis can be found in Github: https://github.com/knights-lab/IMP_analyses.
