# Supplementary material for: Industrialized human gut microbiota increases CD8+ T cells and mucus thickness in humanized mouse gut
Source: Gut Microbes. 2023 Oct 18;15(2):2266627. doi: 10.1080/19490976.2023.2266627 (PMC10588527; doi:10.1080/19490976.2023.2266627)
Supplement: Supplemental Material [file KGMI_A_2266627_SM0757.zip › Supplemental tables and figures/Suppl Table 1.docx]

| **Taxa** | **Donor Type** | **Diet** | **Shortened Taxa** |
| --- | --- | --- | --- |
| k__Bacteria;p__Firmicutes;c__Clostridia;o__Clostridiales;f__Lachnospiraceae;g__Muricomes | 1.54E-04 | 0.12 | Muricomes |
| k__Bacteria;p__Actinobacteria;c__Coriobacteriia;o__Coriobacteriales;f__Coriobacteriaceae;g__Collinsella | 0.017 | 0.59 | Collinsella |
| k__Bacteria;p__Firmicutes;c__Clostridia;o__Clostridiales;f__Ruminococcaceae;g__Negativibacillus | 0.02 | 0.18 | Negativibacillus |
| k__Bacteria;p__Firmicutes;c__Clostridia;o__Clostridiales;f__Ruminococcaceae;g__Subdoligranulum | 0.12 | 2.07E-07 | Subdoligranulum |
| k__Bacteria;p__Firmicutes;c__Clostridia;o__Clostridiales;f__Lachnospiraceae;g__Roseburia | 0.12 | 0.0068 | Roseburia |
| k__Bacteria;p__Proteobacteria;c__Gammaproteobacteria;o__Aeromonadales;f__Succinivibrionaceae;g__Succinatimonas | 0.12 | 0.084 | Succinatimonas |
| k__Bacteria;p__Firmicutes;c__Clostridia;o__Clostridiales;f__Ruminococcaceae;g__Ruminococcus | 0.12 | 0.12 | Ruminococcus |
| k__Bacteria;p__Firmicutes;c__Clostridia;o__Clostridiales;f__Lachnospiraceae | 0.12 | 0.61 | Lachnospiraceae |
| k__Bacteria;p__Actinobacteria;c__Coriobacteriia;o__Eggerthellales;f__Eggerthellaceae;g__Slackia | 0.12 | 0.63 | Slackia |
| k__Bacteria;p__Bacteroidetes;c__Bacteroidia;o__Bacteroidales;f__Rikenellaceae;g__Alistipes | 0.12 | 0.78 | Alistipes |
| k__Bacteria;p__Firmicutes;c__Clostridia;o__Clostridiales;f__;g__Flintibacter | 0.12 | 0.88 | Flintibacter |
| k__Bacteria;p__Proteobacteria;c__Betaproteobacteria;o__Burkholderiales;f__Sutterellaceae;g__Duodenibacillus | 0.13 | 0.16 | Duodenibacillus |
| k__Bacteria;p__Firmicutes;c__Clostridia;o__Clostridiales;f__;g__Pseudoflavonifractor | 0.13 | 0.69 | Pseudoflavonifractor |
| k__Bacteria;p__Firmicutes;c__Clostridia;o__Clostridiales;f__Catabacteriaceae;g__Catabacter | 0.13 | 0.69 | Catabacter |
| k__Bacteria;p__Firmicutes;c__Clostridia;o__Clostridiales;f__Ruminococcaceae;g__Anaerofilum | 0.13 | 0.76 | Anaerofilum |
| k__Bacteria;p__Actinobacteria;c__Coriobacteriia;o__Eggerthellales;f__Eggerthellaceae | 0.13 | 0.88 | Eggerthellaceae |
| k__Bacteria;p__Firmicutes;c__Clostridia;o__Clostridiales;f__Lachnospiraceae;g__Butyrivibrio | 0.13 | 0.88 | Butyrivibrio |
| k__Bacteria;p__Firmicutes;c__Erysipelotrichia;o__Erysipelotrichales;f__Erysipelotrichaceae;g__Erysipelatoclostridium | 0.14 | 0.88 | Erysipelatoclostridium |
| k__Bacteria;p__Actinobacteria;c__Actinobacteria;o__Bifidobacteriales;f__Bifidobacteriaceae;g__Bifidobacterium | 0.14 | 0.92 | Bifidobacterium |
| k__Bacteria;p__Bacteroidetes;c__Bacteroidia;o__Bacteroidales;f__Prevotellaceae;g__Paraprevotella | 0.15 | 0.12 | Paraprevotella |
| k__Bacteria;p__Firmicutes;c__Clostridia;o__Clostridiales;f__Lachnospiraceae;g__Oribacterium | 0.15 | 0.69 | Oribacterium |
| k__Bacteria;p__Firmicutes;c__Clostridia;o__Clostridiales;f__Clostridiaceae;g__Butyricicoccus | 0.16 | 8.53E-04 | Butyricicoccus |
| k__Bacteria;p__Firmicutes;c__Clostridia;o__Clostridiales;f__Lachnospiraceae;g__Anaerosporobacter | 0.16 | 0.06 | Anaerosporobacter |
| k__Bacteria;p__Firmicutes;c__Clostridia;o__Clostridiales;f__Clostridiales Family XIII. Incertae Sedis;g__Ihubacter | 0.16 | 0.08 | Ihubacter |
| k__Bacteria;p__Bacteroidetes;c__Bacteroidia;o__Bacteroidales;f__Bacteroidaceae;g__Bacteroides | 0.16 | 0.14 | Bacteroides |
| k__Bacteria;p__Bacteroidetes;c__Bacteroidia;o__Bacteroidales;f__Prevotellaceae;g__Marseilla | 0.16 | 0.14 | Marseilla |
| k__Bacteria;p__Firmicutes;c__Clostridia;o__Clostridiales;f__Eubacteriaceae;g__Eubacterium | 0.16 | 0.14 | Eubacterium |
| k__Bacteria;p__Lentisphaerae;c__Lentisphaeria;o__Victivallales;f__Victivallaceae;g__Victivallis | 0.16 | 0.22 | Victivallis |
| k__Bacteria | 0.16 | 0.34 | Bacteria |
| k__Bacteria;p__Firmicutes;c__Clostridia;o__Clostridiales;f__Lachnospiraceae;g__Hespellia | 0.16 | 0.47 | Hespellia |
| k__Bacteria;p__Firmicutes;c__Erysipelotrichia;o__Erysipelotrichales;f__Erysipelotrichaceae;g__Faecalitalea | 0.16 | 0.55 | Faecalitalea |
| k__Bacteria;p__Actinobacteria;c__Coriobacteriia | 0.16 | 0.58 | Coriobacteriia |
| k__Bacteria;p__Firmicutes;c__Clostridia;o__Clostridiales;f__Lachnospiraceae;g__Eisenbergiella | 0.16 | 0.96 | Eisenbergiella |
| k__Bacteria;p__Firmicutes;c__Clostridia;o__Clostridiales;f__Peptostreptococcaceae;g__Intestinibacter | 0.18 | 0.14 | Intestinibacter |
| k__Bacteria;p__Firmicutes;c__Clostridia;o__Clostridiales;f__Ruminococcaceae;g__Faecalibacterium | 0.18 | 0.14 | Faecalibacterium |
| k__Bacteria;p__Proteobacteria;c__Gammaproteobacteria;o__Enterobacterales;f__Morganellaceae | 0.18 | 0.27 | Morganellaceae |
| k__Bacteria;p__Firmicutes;c__Clostridia;o__Clostridiales;f__Oscillospiraceae;g__Oscillibacter | 0.18 | 0.43 | Oscillibacter |
| k__Bacteria;p__Firmicutes;c__Clostridia;o__Clostridiales;f__Lachnospiraceae;g__Faecalicatena | 0.18 | 0.43 | Faecalicatena |
| k__Bacteria;p__Proteobacteria;c__Betaproteobacteria;o__Burkholderiales;f__Sutterellaceae;g__Sutterella | 0.18 | 0.7 | Sutterella |
| k__Bacteria;p__Proteobacteria;c__Deltaproteobacteria;o__Desulfovibrionales | 0.18 | 0.86 | Desulfovibrionales |
| k__Bacteria;p__Firmicutes;c__Clostridia;o__Clostridiales;f__Ruminococcaceae;g__Papillibacter | 0.18 | 0.88 | Papillibacter |
| k__Bacteria;p__Proteobacteria;c__Gammaproteobacteria;o__Enterobacterales;f__Erwiniaceae;g__Erwinia | 0.18 | 0.94 | Erwinia |
| k__Bacteria;p__Firmicutes;c__Erysipelotrichia;o__Erysipelotrichales;f__Erysipelotrichaceae;g__Massiliomicrobiota | 0.19 | 0.16 | Massiliomicrobiota |
| k__Bacteria;p__Proteobacteria;c__Betaproteobacteria;o__Burkholderiales;f__Sutterellaceae;g__Parasutterella | 0.19 | 0.39 | Parasutterella |
| k__Bacteria;p__Firmicutes;c__Clostridia;o__Clostridiales;f__Lachnospiraceae;g__Murimonas | 0.19 | 0.69 | Murimonas |
| k__Bacteria;p__Firmicutes;c__Clostridia;o__Clostridiales;f__Lachnospiraceae;g__Blautia | 0.21 | 0.045 | Blautia |
| k__Bacteria;p__Firmicutes;c__Erysipelotrichia;o__Erysipelotrichales;f__Erysipelotrichaceae;g__Faecalicoccus | 0.21 | 0.16 | Faecalicoccus |
| k__Bacteria;p__Verrucomicrobia;c__Verrucomicrobiae;o__Verrucomicrobiales;f__Akkermansiaceae;g__Akkermansia | 0.23 | 0.96 | Akkermansia |
| k__Bacteria;p__Firmicutes;c__Clostridia;o__Clostridiales;f__Ruminococcaceae;g__Gemmiger | 0.25 | 1.51E-03 | Gemmiger |
| k__Bacteria;p__Bacteroidetes;c__Bacteroidia;o__Bacteroidales;f__Muribaculaceae;g__Muribaculum | 0.25 | 0.14 | Muribaculum |
| k__Bacteria;p__Firmicutes;c__Clostridia;o__Clostridiales;f__Ruminococcaceae;g__Drancourtella | 0.25 | 0.6 | Drancourtella |
| k__Bacteria;p__Bacteroidetes;c__Bacteroidia;o__Bacteroidales;f__Tannerellaceae;g__Parabacteroides | 0.25 | 1 | Parabacteroides |
| k__Bacteria;p__Firmicutes;c__Erysipelotrichia;o__Erysipelotrichales;f__Erysipelotrichaceae;g__Holdemania | 0.26 | 0.3 | Holdemania |
| k__Bacteria;p__Firmicutes;c__Erysipelotrichia;o__Erysipelotrichales;f__Erysipelotrichaceae;g__Catenibacterium | 0.3 | 0.13 | Catenibacterium |
| k__Bacteria;p__Firmicutes;c__Negativicutes;o__Veillonellales;f__Veillonellaceae;g__Allisonella | 0.3 | 0.14 | Allisonella |
| k__Bacteria;p__Firmicutes;c__Bacilli;o__Lactobacillales;f__Streptococcaceae;g__Streptococcus | 0.3 | 0.22 | Streptococcus |
| k__Bacteria;p__Firmicutes;c__Clostridia;o__Clostridiales;f__Ruminococcaceae;g__Acutalibacter | 0.3 | 0.47 | Acutalibacter |
| k__Bacteria;p__Actinobacteria;c__Coriobacteriia;o__Coriobacteriales;f__Coriobacteriaceae;g__Enorma | 0.31 | 0.45 | Enorma |
| k__Bacteria;p__Actinobacteria;c__Coriobacteriia;o__Coriobacteriales;f__Coriobacteriaceae;g__Parvibacter | 0.31 | 0.45 | Parvibacter |
| k__Bacteria;p__Firmicutes;c__Clostridia;o__Clostridiales;f__Ruminococcaceae;g__Hydrogenoanaerobacterium | 0.31 | 0.47 | Hydrogenoanaerobacterium |
| k__Bacteria;p__Firmicutes;c__Clostridia;o__Clostridiales;f__Ruminococcaceae;g__Neglecta | 0.34 | 0.12 | Neglecta |
| k__Bacteria;p__Firmicutes;c__Clostridia;o__Clostridiales;f__Peptostreptococcaceae;g__Paraclostridium | 0.34 | 0.45 | Paraclostridium |
| k__Bacteria;p__Firmicutes;c__Clostridia;o__Clostridiales;f__Ruminococcaceae | 0.34 | 0.47 | Ruminococcaceae |
| k__Bacteria;p__Proteobacteria;c__Betaproteobacteria;o__Burkholderiales;f__Oxalobacteraceae;g__Oxalobacter | 0.34 | 0.47 | Oxalobacter |
| k__Bacteria;p__Firmicutes;c__Erysipelotrichia;o__Erysipelotrichales;f__Erysipelotrichaceae;g__Holdemanella | 0.35 | 0.39 | Holdemanella |
| k__Bacteria;p__Firmicutes;c__Clostridia;o__Clostridiales;f__Lachnospiraceae;g__Lachnospira | 0.37 | 0.15 | Lachnospira |
| k__Bacteria;p__Firmicutes;c__Erysipelotrichia;o__Erysipelotrichales;f__Erysipelotrichaceae;g__Coprobacillus | 0.37 | 0.22 | Coprobacillus |
| k__Bacteria;p__Firmicutes;c__Clostridia;o__Clostridiales;f__;g__Flavonifractor | 0.38 | 0.034 | Flavonifractor |
| k__Bacteria;p__Firmicutes;c__Clostridia;o__Clostridiales;f__Ruminococcaceae;g__Caproiciproducens | 0.38 | 0.16 | Caproiciproducens |
| k__Bacteria;p__Firmicutes;c__Clostridia;o__Clostridiales;f__Ruminococcaceae;g__Anaeromassilibacillus | 0.38 | 0.23 | Anaeromassilibacillus |
| k__Bacteria;p__Firmicutes;c__Clostridia;o__Clostridiales;f__Ruminococcaceae;g__Phocea | 0.38 | 0.43 | Phocea |
| k__Bacteria;p__Bacteroidetes;c__Bacteroidia;o__Bacteroidales;f__Prevotellaceae;g__Massiliprevotella | 0.38 | 0.58 | Massiliprevotella |
| k__Bacteria;p__Firmicutes;c__Clostridia;o__Clostridiales;f__Lachnospiraceae;g__Extibacter | 0.38 | 0.58 | Extibacter |
| k__Bacteria;p__Firmicutes;c__Clostridia;o__Clostridiales;f__Clostridiales Family XIII. Incertae Sedis | 0.38 | 0.78 | Clostridiales Family XIII. Incertae Sedis |
| k__Bacteria;p__Firmicutes;c__Clostridia;o__Clostridiales;f__Peptostreptococcaceae;g__Paeniclostridium | 0.39 | 0.12 | Paeniclostridium |
| k__Bacteria;p__Firmicutes;c__Negativicutes;o__Selenomonadales;f__Selenomonadaceae;g__Mitsuokella | 0.39 | 0.14 | Mitsuokella |
| k__Bacteria;p__Actinobacteria;c__Coriobacteriia;o__Coriobacteriales;f__Coriobacteriaceae;g__Senegalimassilia | 0.39 | 0.25 | Senegalimassilia |
| k__Bacteria;p__Firmicutes;c__Negativicutes;o__Veillonellales;f__Veillonellaceae;g__Veillonella | 0.39 | 0.62 | Veillonella |
| k__Bacteria;p__Actinobacteria;c__Coriobacteriia;o__Coriobacteriales;f__Atopobiaceae;g__Olsenella | 0.39 | 0.94 | Olsenella |
| k__Bacteria;p__Firmicutes | 0.4 | 0.14 | Firmicutes |
| k__Bacteria;p__Proteobacteria;c__Betaproteobacteria;o__Burkholderiales;f__Oxalobacteraceae;g__Herbaspirillum | 0.4 | 0.86 | Herbaspirillum |
| k__Bacteria;p__Actinobacteria;c__Coriobacteriia;o__Eggerthellales;f__Eggerthellaceae;g__Enterorhabdus | 0.4 | 0.86 | Enterorhabdus |
| k__Bacteria;p__Firmicutes;c__Clostridia;o__Clostridiales;f__Ruminococcaceae;g__Ruminiclostridium | 0.41 | 0.22 | Ruminiclostridium |
| k__Bacteria;p__Firmicutes;c__Clostridia;o__Clostridiales;f__Ruminococcaceae;g__Ethanoligenens | 0.41 | 0.4 | Ethanoligenens |
| k__Bacteria;p__Firmicutes;c__Bacilli;o__Lactobacillales;f__Lactobacillaceae;g__Lactobacillus | 0.41 | 0.84 | Lactobacillus |
| k__Bacteria;p__Firmicutes;c__Negativicutes;o__Veillonellales;f__Veillonellaceae;g__Dialister | 0.42 | 0.18 | Dialister |
| k__Bacteria;p__Firmicutes;c__Clostridia;o__Clostridiales;f__Clostridiaceae | 0.43 | 0.14 | Clostridiaceae |
| k__Bacteria;p__Firmicutes;c__Clostridia;o__Clostridiales;f__Ruminococcaceae;g__Sporobacter | 0.43 | 0.21 | Sporobacter |
| k__Bacteria;p__Bacteroidetes;c__Bacteroidia;o__Bacteroidales;f__Odoribacteraceae;g__Odoribacter | 0.43 | 0.66 | Odoribacter |
| k__Bacteria;p__Firmicutes;c__Bacilli;o__Bacillales;f__Listeriaceae;g__Listeria | 0.45 | 0.16 | Listeria |
| k__Bacteria;p__Firmicutes;c__Clostridia;o__Clostridiales;f__Peptostreptococcaceae;g__Clostridioides | 0.45 | 0.48 | Clostridioides |
| k__Bacteria;p__Firmicutes;c__Erysipelotrichia;o__Erysipelotrichales;f__Erysipelotrichaceae;g__Dielma | 0.45 | 0.58 | Dielma |
| k__Bacteria;p__Firmicutes;c__Clostridia;o__Clostridiales;f__Clostridiaceae;g__Hungatella | 0.45 | 0.88 | Hungatella |
| k__Bacteria;p__Firmicutes;c__Clostridia;o__Clostridiales;f__Lachnospiraceae;g__Robinsoniella | 0.45 | 0.88 | Robinsoniella |
| k__Bacteria;p__Firmicutes;c__Negativicutes;o__Selenomonadales;f__Selenomonadaceae;g__Megamonas | 0.47 | 0.41 | Megamonas |
| k__Bacteria;p__Proteobacteria;c__Gammaproteobacteria;o__Enterobacterales | 0.5 | 0.38 | Enterobacterales |
| k__Bacteria;p__Firmicutes;c__Clostridia;o__Clostridiales;f__Lachnospiraceae;g__Anaerobium | 0.5 | 0.7 | Anaerobium |
| k__Bacteria;p__Bacteroidetes;c__Bacteroidia;o__Bacteroidales | 0.5 | 0.96 | Bacteroidales |
| k__Bacteria;p__Firmicutes;c__Erysipelotrichia;o__Erysipelotrichales;f__Erysipelotrichaceae | 0.52 | 0.22 | Erysipelotrichaceae |
| k__Bacteria;p__Firmicutes;c__Clostridia;o__Clostridiales;f__Lachnospiraceae;g__Cuneatibacter | 0.53 | 0.14 | Cuneatibacter |
| k__Bacteria;p__Proteobacteria;c__Gammaproteobacteria;o__Enterobacterales;f__Enterobacteriaceae;g__Kluyvera | 0.53 | 0.71 | Kluyvera |
| k__Bacteria;p__Proteobacteria;c__Gammaproteobacteria;o__Enterobacterales;f__Morganellaceae;g__Proteus | 0.53 | 0.71 | Proteus |
| k__Bacteria;p__Proteobacteria;c__Deltaproteobacteria;o__Desulfovibrionales;f__Desulfovibrionaceae;g__Desulfovibrio | 0.54 | 0.29 | Desulfovibrio |
| k__Bacteria;p__Bacteroidetes;c__Bacteroidia;o__Bacteroidales;f__Odoribacteraceae;g__Butyricimonas | 0.58 | 0.58 | Butyricimonas |
| k__Bacteria;p__Firmicutes;c__Clostridia;o__Clostridiales;f__Clostridiales Family XIII. Incertae Sedis;g__Emergencia | 0.58 | 0.61 | Emergencia |
| k__Bacteria;p__Firmicutes;c__Clostridia;o__Clostridiales;f__Lachnospiraceae;g__Coprococcus | 0.6 | 0.56 | Coprococcus |
| k__Bacteria;p__Actinobacteria;c__Coriobacteriia;o__Eggerthellales;f__Eggerthellaceae;g__Eggerthella | 0.62 | 0.088 | Eggerthella |
| k__Bacteria;p__Firmicutes;c__Clostridia;o__Clostridiales;f__Christensenellaceae;g__Christensenella | 0.62 | 0.14 | Christensenella |
| k__Bacteria;p__Firmicutes;c__Clostridia;o__Clostridiales;f__Ruminococcaceae;g__Acetivibrio | 0.62 | 0.93 | Acetivibrio |
| k__Bacteria;p__Firmicutes;c__Clostridia;o__Clostridiales;f__Lachnospiraceae;g__Tyzzerella | 0.62 | 0.96 | Tyzzerella |
| k__Bacteria;p__Actinobacteria;c__Coriobacteriia;o__Eggerthellales;f__Eggerthellaceae;g__Raoultibacter | 0.63 | 0.17 | Raoultibacter |
| k__Bacteria;p__Bacteroidetes;c__Bacteroidia;o__Bacteroidales;f__Prevotellaceae;g__Prevotella | 0.63 | 0.38 | Prevotella |
| k__Bacteria;p__Proteobacteria;c__Gammaproteobacteria;o__Enterobacterales;f__Morganellaceae;g__Morganella | 0.63 | 0.43 | Morganella |
| k__Bacteria;p__Firmicutes;c__Clostridia;o__Clostridiales;f__Eubacteriaceae;g__Anaerofustis | 0.63 | 0.7 | Anaerofustis |
| k__Bacteria;p__Firmicutes;c__Clostridia;o__Clostridiales;f__Lachnospiraceae;g__Anaerocolumna | 0.65 | 0.94 | Anaerocolumna |
| k__Bacteria;p__Bacteroidetes;c__Bacteroidia;o__Bacteroidales;f__Barnesiellaceae;g__Barnesiella | 0.67 | 0.5 | Barnesiella |
| k__Bacteria;p__Firmicutes;c__Erysipelotrichia;o__Erysipelotrichales;f__Erysipelotrichaceae;g__Turicibacter | 0.75 | 0.26 | Turicibacter |
| k__Bacteria;p__Firmicutes;c__Clostridia;o__Clostridiales;f__Peptostreptococcaceae | 0.75 | 0.33 | Peptostreptococcaceae |
| k__Bacteria;p__Proteobacteria;c__Gammaproteobacteria;o__Enterobacterales;f__Pectobacteriaceae;g__Pectobacterium | 0.75 | 0.46 | Pectobacterium |
| k__Bacteria;p__Firmicutes;c__Bacilli;o__Bacillales;f__Staphylococcaceae;g__Staphylococcus | 0.75 | 0.94 | Staphylococcus |
| k__Bacteria;p__Firmicutes;c__Bacilli;o__Lactobacillales;f__Streptococcaceae;g__Lactococcus | 0.76 | 1.51E-03 | Lactococcus |
| k__Bacteria;p__Actinobacteria;c__Actinobacteria;o__Actinomycetales;f__Actinomycetaceae;g__Actinomyces | 0.76 | 0.21 | Actinomyces |
| k__Bacteria;p__Bacteroidetes;c__Bacteroidia;o__Bacteroidales;f__Prevotellaceae;g__Prevotellamassilia | 0.76 | 0.26 | Prevotellamassilia |
| k__Bacteria;p__Firmicutes;c__Negativicutes;o__Acidaminococcales;f__Acidaminococcaceae;g__Phascolarctobacterium | 0.76 | 0.29 | Phascolarctobacterium |
| k__Bacteria;p__Firmicutes;c__Clostridia;o__Clostridiales;f__Clostridiaceae;g__Mordavella | 0.76 | 0.7 | Mordavella |
| k__Bacteria;p__Firmicutes;c__Clostridia;o__Clostridiales;f__Eubacteriaceae;g__Intestinibacillus | 0.76 | 0.91 | Intestinibacillus |
| k__Bacteria;p__Firmicutes;c__Bacilli;o__Bacillales;f__Bacillaceae;g__Lysinibacillus | 0.79 | 0.88 | Lysinibacillus |
| k__Bacteria;p__Firmicutes;c__Clostridia;o__Clostridiales;f__Peptostreptococcaceae;g__Romboutsia | 0.81 | 5.66E-06 | Romboutsia |
| k__Bacteria;p__Firmicutes;c__Erysipelotrichia;o__Erysipelotrichales;f__Erysipelotrichaceae;g__Bulleidia | 0.81 | 0.39 | Bulleidia |
| k__Bacteria;p__Firmicutes;c__Clostridia;o__Clostridiales;f__;g__Intestinimonas | 0.81 | 0.69 | Intestinimonas |
| k__Bacteria;p__Fusobacteria;c__Fusobacteriia;o__Fusobacteriales;f__Fusobacteriaceae;g__Fusobacterium | 0.84 | 0.88 | Fusobacterium |
| k__Bacteria;p__Firmicutes;c__Clostridia;o__Clostridiales;f__Lachnospiraceae;g__Dorea | 0.88 | 0.022 | Dorea |
| k__Bacteria;p__Firmicutes;c__Bacilli;o__Lactobacillales;f__Enterococcaceae;g__Enterococcus | 0.88 | 0.033 | Enterococcus |
| k__Bacteria;p__Firmicutes;c__Clostridia;o__Clostridiales;f__Peptostreptococcaceae;g__Terrisporobacter | 0.88 | 0.06 | Terrisporobacter |
| k__Bacteria;p__Proteobacteria;c__Alphaproteobacteria;o__Rhizobiales;f__Bradyrhizobiaceae;g__Bradyrhizobium | 0.88 | 0.12 | Bradyrhizobium |
| k__Bacteria;p__Actinobacteria;c__Coriobacteriia;o__Eggerthellales;f__Eggerthellaceae;g__Gordonibacter | 0.88 | 0.17 | Gordonibacter |
| k__Bacteria;p__Firmicutes;c__Clostridia;o__Clostridiales;f__Lachnospiraceae;g__Anaerostipes | 0.88 | 0.17 | Anaerostipes |
| k__Bacteria;p__Firmicutes;c__Clostridia;o__Clostridiales;f__Lachnospiraceae;g__Lachnoclostridium | 0.88 | 0.19 | Lachnoclostridium |
| k__Bacteria;p__Proteobacteria;c__Gammaproteobacteria;o__Pasteurellales;f__Pasteurellaceae;g__Haemophilus | 0.88 | 0.28 | Haemophilus |
| k__Bacteria;p__Firmicutes;c__Erysipelotrichia;o__Erysipelotrichales;f__Erysipelotrichaceae;g__Longibaculum | 0.88 | 0.55 | Longibaculum |
| k__Bacteria;p__Proteobacteria;c__Gammaproteobacteria;o__Enterobacterales;f__Enterobacteriaceae;g__Shigella | 0.88 | 0.84 | Shigella |
| k__Bacteria;p__Firmicutes;c__Clostridia;o__Clostridiales;f__;g__Howardella | 0.88 | 0.86 | Howardella |
| k__Bacteria;p__Proteobacteria;c__Gammaproteobacteria;o__Enterobacterales;f__Enterobacteriaceae | 0.88 | 0.88 | Enterobacteriaceae |
| k__Bacteria;p__Proteobacteria;c__Betaproteobacteria;o__Burkholderiales;f__Oxalobacteraceae | 0.88 | 0.9 | Oxalobacteraceae |
| k__Bacteria;p__Bacteroidetes;c__Flavobacteriia;o__Flavobacteriales;f__Flavobacteriaceae;g__Chryseobacterium | 0.9 | 0.58 | Chryseobacterium |
| k__Bacteria;p__Firmicutes;c__Clostridia;o__Clostridiales;f__Clostridiaceae;g__Clostridium | 0.92 | 0.14 | Clostridium |
| k__Bacteria;p__Firmicutes;c__Clostridia;o__Clostridiales;f__Ruminococcaceae;g__Anaerotruncus | 0.95 | 0.14 | Anaerotruncus |
| k__Bacteria;p__Firmicutes;c__Negativicutes;o__Veillonellales;f__Veillonellaceae;g__Megasphaera | 0.95 | 0.47 | Megasphaera |
| k__Bacteria;p__Firmicutes;c__Clostridia;o__Clostridiales;f__Lachnospiraceae;g__Fusicatenibacter | 0.95 | 0.84 | Fusicatenibacter |
| k__Bacteria;p__Actinobacteria;c__Actinobacteria;o__Corynebacteriales;f__Corynebacteriaceae;g__Corynebacterium | 0.96 | 0.84 | Corynebacterium |
| k__Bacteria;p__Firmicutes;c__Clostridia;o__Clostridiales | 0.96 | 0.88 | Clostridiales |
| k__Bacteria;p__Firmicutes;c__Clostridia;o__Clostridiales;f__Clostridiaceae;g__Lactonifactor | 0.96 | 0.93 | Lactonifactor |
| k__Bacteria;p__Actinobacteria;c__Actinobacteria;o__Propionibacteriales;f__Propionibacteriaceae;g__Cutibacterium | 0.98 | 0.58 | Cutibacterium |
| k__Bacteria;p__Firmicutes;c__Clostridia;o__Clostridiales;f__Lachnospiraceae;g__Frisingicoccus | 0.99 | 0.69 | Frisingicoccus |
