# Supplementary material for: Industrialized human gut microbiota increases CD8+ T cells and mucus thickness in humanized mouse gut
Source: Gut Microbes. 2023 Oct 18;15(2):2266627. doi: 10.1080/19490976.2023.2266627 (PMC10588527; doi:10.1080/19490976.2023.2266627)
Supplement: Supplemental Material [file KGMI_A_2266627_SM0757.zip › Supplemental tables and figures/Suppl Table 2.docx]

| Mouse ID | Body Weight (g) | Fasting Glucose | Donor | Donor Type | Cohoused | Cage ID | Diet | Diet Type | Food Weight Out (g) | Food Weight In (g) | Date |
| --- | --- | --- | --- | --- | --- | --- | --- | --- | --- | --- | --- |
| M1 | 20.3 | 124 | TFSCS023 | Thai | FALSE | 1 | TD.86489 | LowFiber | NA | 227.4 | 7/11/17 |
| M2 | 18.7 | 218 | TFSCS023 | Thai | FALSE | 1 | TD.86489 | LowFiber | NA | 227.4 | 7/11/17 |
| M3 | 16.8 | 167 | TFSCS023 | Thai | FALSE | 1 | TD.86489 | LowFiber | NA | 227.4 | 7/11/17 |
| M4 | 15.9 | 113 | TFSCS023 | Thai | FALSE | 1 | TD.86489 | LowFiber | NA | 227.4 | 7/11/17 |
| M5 | 16.7 | 155 | TFSCS023 | Thai | FALSE | 1 | TD.86489 | LowFiber | NA | 227.4 | 7/11/17 |
| M6 | 18 | 98 | IMP.263 | US | FALSE | 2 | TD.86489 | LowFiber | NA | 226.8 | 7/11/17 |
| M7 | 18.5 | 126 | IMP.263 | US | FALSE | 2 | TD.86489 | LowFiber | NA | 226.8 | 7/11/17 |
| M8 | 17.3 | 99 | IMP.263 | US | FALSE | 2 | TD.86489 | LowFiber | NA | 226.8 | 7/11/17 |
| M9 | 18.6 | 104 | IMP.263 | US | FALSE | 2 | TD.86489 | LowFiber | NA | 226.8 | 7/11/17 |
| M10 | 19.4 | 148 | IMP.263 | US | FALSE | 2 | TD.86489 | LowFiber | NA | 226.8 | 7/11/17 |
| M1 | 20.7 | NA | TFSCS023 | Thai | FALSE | 1 | TD.86489 | LowFiber | 14.3 | 228.8 | 7/25/17 |
| M2 | 19.7 | NA | TFSCS023 | Thai | FALSE | 1 | TD.86489 | LowFiber | 14.3 | 228.8 | 7/25/17 |
| M3 | 18.1 | NA | TFSCS023 | Thai | FALSE | 1 | TD.86489 | LowFiber | 14.3 | 228.8 | 7/25/17 |
| M4 | 17.8 | NA | TFSCS023 | Thai | FALSE | 1 | TD.86489 | LowFiber | 14.3 | 228.8 | 7/25/17 |
| M5 | 18.2 | NA | TFSCS023 | Thai | FALSE | 1 | TD.86489 | LowFiber | 14.3 | 228.8 | 7/25/17 |
| M6 | 20.9 | NA | IMP.263 | US | FALSE | 2 | TD.86489 | LowFiber | 7.1 | 227.9 | 7/25/17 |
| M7 | 20 | NA | IMP.263 | US | FALSE | 2 | TD.86489 | LowFiber | 7.1 | 227.9 | 7/25/17 |
| M8 | 19.4 | NA | IMP.263 | US | FALSE | 2 | TD.86489 | LowFiber | 7.1 | 227.9 | 7/25/17 |
| M9 | 20.6 | NA | IMP.263 | US | FALSE | 2 | TD.86489 | LowFiber | 7.1 | 227.9 | 7/25/17 |
| M10 | 20.3 | NA | IMP.263 | US | FALSE | 2 | TD.86489 | LowFiber | 7.1 | 227.9 | 7/25/17 |
| M1 | 21.4 | NA | TFSCS023 | Thai | FALSE | 1 | TD.86489 | LowFiber | 29.9 | 206 | 8/8/17 |
| M2 | 20.3 | NA | TFSCS023 | Thai | FALSE | 1 | TD.86489 | LowFiber | 29.9 | 206 | 8/8/17 |
| M3 | 19 | NA | TFSCS023 | Thai | FALSE | 1 | TD.86489 | LowFiber | 29.9 | 206 | 8/8/17 |
| M4 | 18.4 | NA | TFSCS023 | Thai | FALSE | 1 | TD.86489 | LowFiber | 29.9 | 206 | 8/8/17 |
| M5 | 19.5 | NA | TFSCS023 | Thai | FALSE | 1 | TD.86489 | LowFiber | 29.9 | 206 | 8/8/17 |
| M6 | 20.1 | NA | IMP.263 | US | FALSE | 2 | TD.86489 | LowFiber | 37.1 | 214.7 | 8/8/17 |
| M7 | 19.1 | NA | IMP.263 | US | FALSE | 2 | TD.86489 | LowFiber | 37.1 | 214.7 | 8/8/17 |
| M8 | 19.7 | NA | IMP.263 | US | FALSE | 2 | TD.86489 | LowFiber | 37.1 | 214.7 | 8/8/17 |
| M9 | 20.1 | NA | IMP.263 | US | FALSE | 2 | TD.86489 | LowFiber | 37.1 | 214.7 | 8/8/17 |
| M10 | 21.3 | NA | IMP.263 | US | FALSE | 2 | TD.86489 | LowFiber | 37.1 | 214.7 | 8/8/17 |
| M1 | 21.8 | NA | TFSCS023 | Thai | FALSE | 1 | TD.86489 | LowFiber | 7.4 | 229 | 8/22/17 |
| M2 | 21.5 | NA | TFSCS023 | Thai | FALSE | 1 | TD.86489 | LowFiber | 7.4 | 229 | 8/22/17 |
| M3 | 20.8 | NA | TFSCS023 | Thai | FALSE | 1 | TD.86489 | LowFiber | 7.4 | 229 | 8/22/17 |
| M4 | 21.1 | NA | TFSCS023 | Thai | FALSE | 1 | TD.86489 | LowFiber | 7.4 | 229 | 8/22/17 |
| M5 | 22.4 | NA | TFSCS023 | Thai | FALSE | 1 | TD.86489 | LowFiber | 7.4 | 229 | 8/22/17 |
| M6 | 20.9 | NA | IMP.263 | US | FALSE | 2 | TD.86489 | LowFiber | 2 | 403.4 | 8/22/17 |
| M7 | 22.3 | NA | IMP.263 | US | FALSE | 2 | TD.86489 | LowFiber | 2 | 403.4 | 8/22/17 |
| M8 | 19.7 | NA | IMP.263 | US | FALSE | 2 | TD.86489 | LowFiber | 2 | 403.4 | 8/22/17 |
| M9 | 20.5 | NA | IMP.263 | US | FALSE | 2 | TD.86489 | LowFiber | 2 | 403.4 | 8/22/17 |
| M10 | 21.6 | NA | IMP.263 | US | FALSE | 2 | TD.86489 | LowFiber | 2 | 403.4 | 8/22/17 |
| M1 | 22.1 | NA | TFSCS023 | Thai | FALSE | 1 | TD.86489 | LowFiber | 34.9 | 197.1 | 9/5/17 |
| M2 | 22.7 | NA | TFSCS023 | Thai | FALSE | 1 | TD.86489 | LowFiber | 34.9 | 197.1 | 9/5/17 |
| M3 | 20.2 | 365 | TFSCS023 | Thai | FALSE | 1 | TD.86489 | LowFiber | 34.9 | NA | 9/5/17 |
| M4 | 22.9 | 362 | TFSCS023 | Thai | FALSE | 1 | TD.86489 | LowFiber | 34.9 | NA | 9/5/17 |
| M5 | 22.8 | 221 | TFSCS023 | Thai | FALSE | 1 | TD.86489 | LowFiber | 34.9 | NA | 9/5/17 |
| M6 | 23.7 | 277 | IMP.263 | US | FALSE | 2 | TD.86489 | LowFiber | 162.2 | NA | 9/5/17 |
| M7 | 21.5 | 144 | IMP.263 | US | FALSE | 2 | TD.86489 | LowFiber | 162.2 | NA | 9/5/17 |
| M8 | 21.4 | NA | IMP.263 | US | FALSE | 2 | TD.86489 | LowFiber | 162.2 | 197.1 | 9/5/17 |
| M9 | 23.9 | NA | IMP.263 | US | FALSE | 2 | TD.86489 | LowFiber | 162.2 | 197.1 | 9/5/17 |
| M10 | 22.9 | 382 | IMP.263 | US | FALSE | 2 | TD.86489 | LowFiber | 162.2 | NA | 9/5/17 |
| M1 | 23.2 | 324 | TFSCS023 | Thai | TRUE | C1-2 | TD.86489 | LowFiber | 6.5 | NA | 9/19/17 |
| M2 | 24.2 | 188 | TFSCS023 | Thai | TRUE | C1-2 | TD.86489 | LowFiber | 6.5 | NA | 9/19/17 |
| M8 | 21.8 | 319 | IMP.263 | US | TRUE | C1-2 | TD.86489 | LowFiber | 6.5 | NA | 9/19/17 |
| M9 | 24.2 | 263 | IMP.263 | US | TRUE | C1-2 | TD.86489 | LowFiber | 6.5 | NA | 9/19/17 |
| M11 | 14.5 | 183 | TFSCS023 | Thai | FALSE | 5 | LabDiet 5061 | HighFiber | NA | 93.2 | 10/3/17 |
| M12 | 18.8 | 187 | TFSCS023 | Thai | FALSE | 3 | LabDiet 5061 | HighFiber | NA | 266.1 | 10/3/17 |
| M13 | 13.8 | 129 | TFSCS023 | Thai | FALSE | 3 | LabDiet 5061 | HighFiber | NA | 266.1 | 10/3/17 |
| M14 | 20.3 | 131 | TFSCS023 | Thai | FALSE | 3 | LabDiet 5061 | HighFiber | NA | 266.1 | 10/3/17 |
| M15 | 15.6 | 128 | TFSCS023 | Thai | FALSE | 3 | LabDiet 5061 | HighFiber | NA | 266.1 | 10/3/17 |
| M16 | 12.7 | 117 | IMP.263 | US | FALSE | 4 | LabDiet 5061 | HighFiber | NA | 251.9 | 10/3/17 |
| M17 | 15.7 | 118 | IMP.263 | US | FALSE | 4 | LabDiet 5061 | HighFiber | NA | 251.9 | 10/3/17 |
| M18 | 17.3 | 102 | IMP.263 | US | FALSE | 4 | LabDiet 5061 | HighFiber | NA | 251.9 | 10/3/17 |
| M19 | 20 | 142 | IMP.263 | US | FALSE | 4 | LabDiet 5061 | HighFiber | NA | 251.9 | 10/3/17 |
| M20 | 19.3 | 131 | IMP.263 | US | FALSE | 4 | LabDiet 5061 | HighFiber | NA | 251.9 | 10/3/17 |
| M11 | 17.1 | NA | TFSCS023 | Thai | FALSE | 5 | LabDiet 5061 | HighFiber | 44.8 | 168.9 | 10/17/17 |
| M12 | 20.6 | NA | TFSCS023 | Thai | FALSE | 3 | LabDiet 5061 | HighFiber | 43.2 | 286.6 | 10/17/17 |
| M13 | 15.9 | NA | TFSCS023 | Thai | FALSE | 3 | LabDiet 5061 | HighFiber | 43.2 | 286.6 | 10/17/17 |
| M14 | 21.6 | NA | TFSCS023 | Thai | FALSE | 3 | LabDiet 5061 | HighFiber | 43.2 | 286.6 | 10/17/17 |
| M15 | 18.5 | NA | TFSCS023 | Thai | FALSE | 3 | LabDiet 5061 | HighFiber | 43.2 | 286.6 | 10/17/17 |
| M16 | 11.7 | NA | IMP.263 | US | FALSE | 4 | LabDiet 5061 | HighFiber | 3.2 | 294.8 | 10/17/17 |
| M17 | 15.1 | NA | IMP.263 | US | FALSE | 4 | LabDiet 5061 | HighFiber | 3.2 | 294.8 | 10/17/17 |
| M18 | 17.2 | NA | IMP.263 | US | FALSE | 4 | LabDiet 5061 | HighFiber | 3.2 | 294.8 | 10/17/17 |
| M19 | 19.5 | NA | IMP.263 | US | FALSE | 4 | LabDiet 5061 | HighFiber | 3.2 | 294.8 | 10/17/17 |
| M20 | 18.9 | NA | IMP.263 | US | FALSE | 4 | LabDiet 5061 | HighFiber | 3.2 | 294.8 | 10/17/17 |
| M11 | 19.8 | NA | TFSCS023 | Thai | FALSE | 5 | LabDiet 5061 | HighFiber | 113.9 | 113.9 | 10/31/17 |
| M12 | 21.1 | NA | TFSCS023 | Thai | FALSE | 3 | LabDiet 5061 | HighFiber | 81.9 | 449.1 | 10/31/17 |
| M13 | 17 | NA | TFSCS023 | Thai | FALSE | 3 | LabDiet 5061 | HighFiber | 81.9 | 449.1 | 10/31/17 |
| M14 | 21.9 | NA | TFSCS023 | Thai | FALSE | 3 | LabDiet 5061 | HighFiber | 81.9 | 449.1 | 10/31/17 |
| M15 | 19.8 | NA | TFSCS023 | Thai | FALSE | 3 | LabDiet 5061 | HighFiber | 81.9 | 449.1 | 10/31/17 |
| M16 | 16.5 | NA | IMP.263 | US | FALSE | 4 | LabDiet 5061 | HighFiber | 35 | 474.6 | 10/31/17 |
| M17 | 18.1 | NA | IMP.263 | US | FALSE | 4 | LabDiet 5061 | HighFiber | 35 | 474.6 | 10/31/17 |
| M18 | 19.7 | NA | IMP.263 | US | FALSE | 4 | LabDiet 5061 | HighFiber | 35 | 474.6 | 10/31/17 |
| M19 | 23.3 | NA | IMP.263 | US | FALSE | 4 | LabDiet 5061 | HighFiber | 35 | 474.6 | 10/31/17 |
| M20 | 20.7 | NA | IMP.263 | US | FALSE | 4 | LabDiet 5061 | HighFiber | 35 | 474.6 | 10/31/17 |
| M11 | 23.2 | NA | TFSCS023 | Thai | FALSE | 5 | LabDiet 5061 | HighFiber | 62.2 | 238.8 | 11/14/17 |
| M12 | 22.3 | NA | TFSCS023 | Thai | FALSE | 3 | LabDiet 5061 | HighFiber | 157.7 | 309.5 | 11/14/17 |
| M13 | 18.4 | NA | TFSCS023 | Thai | FALSE | 3 | LabDiet 5061 | HighFiber | 157.7 | 231.6 | 11/14/17 |
| M14 | 23.3 | NA | TFSCS023 | Thai | FALSE | 3 | LabDiet 5061 | HighFiber | 157.7 | 231.6 | 11/14/17 |
| M15 | 20.8 | NA | TFSCS023 | Thai | FALSE | 3 | LabDiet 5061 | HighFiber | 157.7 | 309.5 | 11/14/17 |
| M16 | 17.1 | NA | IMP.263 | US | FALSE | 4 | LabDiet 5061 | HighFiber | 138.6 | 233.9 | 11/14/17 |
| M17 | 19.1 | NA | IMP.263 | US | FALSE | 4 | LabDiet 5061 | HighFiber | 138.6 | 233.9 | 11/14/17 |
| M18 | 20.2 | NA | IMP.263 | US | FALSE | 4 | LabDiet 5061 | HighFiber | 138.6 | 314.8 | 11/14/17 |
| M19 | 22.6 | NA | IMP.263 | US | FALSE | 4 | LabDiet 5061 | HighFiber | 138.6 | 314.8 | 11/14/17 |
| M20 | 22.4 | NA | IMP.263 | US | FALSE | 4 | LabDiet 5061 | HighFiber | 138.6 | 233.9 | 11/14/17 |
| M11 | 20.8 | 169 | TFSCS023 | Thai | FALSE | 5 | LabDiet 5061 | HighFiber | 165.3 | NA | 11/28/17 |
| M12 | 22.4 | NA | TFSCS023 | Thai | FALSE | 3 | LabDiet 5061 | HighFiber | 96 | 369.9 | 11/28/17 |
| M13 | 18.7 | 143 | TFSCS023 | Thai | FALSE | 3 | LabDiet 5061 | HighFiber | 135.6 | NA | 11/28/17 |
| M14 | 23.2 | 176 | TFSCS023 | Thai | FALSE | 3 | LabDiet 5061 | HighFiber | 135.6 | NA | 11/28/17 |
| M15 | 22.1 | NA | TFSCS023 | Thai | FALSE | 3 | LabDiet 5061 | HighFiber | 96 | 369.9 | 11/28/17 |
| M16 | 17.8 | 113 | IMP.263 | US | FALSE | 4 | LabDiet 5061 | HighFiber | 167.6 | NA | 11/28/17 |
| M17 | 20.6 | 240 | IMP.263 | US | FALSE | 4 | LabDiet 5061 | HighFiber | 167.6 | NA | 11/28/17 |
| M18 | 22.2 | NA | IMP.263 | US | FALSE | 4 | LabDiet 5061 | HighFiber | 66.3 | 369.9 | 11/28/17 |
| M19 | 25.2 | NA | IMP.263 | US | FALSE | 4 | LabDiet 5061 | HighFiber | 66.3 | 369.9 | 11/28/17 |
| M20 | 22.7 | 157 | IMP.263 | US | FALSE | 4 | LabDiet 5061 | HighFiber | 167.6 | NA | 11/28/17 |
| M12 | 23.5 | 190 | TFSCS023 | Thai | TRUE | C3-4 | LabDiet 5061 | HighFiber | 149.6 | NA | 12/12/17 |
| M15 | 21.7 | 112 | TFSCS023 | Thai | TRUE | C3-4 | LabDiet 5061 | HighFiber | 149.6 | NA | 12/12/17 |
| M18 | 21.6 | 177 | IMP.263 | US | TRUE | C3-4 | LabDiet 5061 | HighFiber | 149.6 | NA | 12/12/17 |
| M19 | 24.7 | 155 | IMP.263 | US | TRUE | C3-4 | LabDiet 5061 | HighFiber | 149.6 | NA | 12/12/17 |
| M21 | 11.5 | 126 | TFSCS026 | Thai | FALSE | 6 | TD.86489 | LowFiber | NA | 233.3 | 10/25/17 |
| M22 | 16.6 | 128 | TFSCS026 | Thai | FALSE | 6 | TD.86489 | LowFiber | NA | 233.3 | 10/25/17 |
| M23 | 17.4 | 144 | TFSCS026 | Thai | FALSE | 6 | TD.86489 | LowFiber | NA | 233.3 | 10/25/17 |
| M24 | 16.6 | 111 | TFSCS026 | Thai | FALSE | 6 | TD.86489 | LowFiber | NA | 233.3 | 10/25/17 |
| M25 | 16.8 | 102 | TFSCS026 | Thai | FALSE | 6 | TD.86489 | LowFiber | NA | 233.3 | 10/25/17 |
| M26 | 14.7 | 114 | IMP.264 | US | FALSE | 7 | TD.86489 | LowFiber | NA | 221.4 | 10/25/17 |
| M27 | 15.6 | 73 | IMP.264 | US | FALSE | 7 | TD.86489 | LowFiber | NA | 221.4 | 10/25/17 |
| M28 | 16.8 | 103 | IMP.264 | US | FALSE | 7 | TD.86489 | LowFiber | NA | 221.4 | 10/25/17 |
| M29 | 15.9 | 80 | IMP.264 | US | FALSE | 7 | TD.86489 | LowFiber | NA | 221.4 | 10/25/17 |
| M30 | 16.1 | 125 | IMP.264 | US | FALSE | 7 | TD.86489 | LowFiber | NA | 221.4 | 10/25/17 |
| M21 | 17.6 | NA | TFSCS026 | Thai | FALSE | 6 | TD.86489 | LowFiber | 14.6 | 208 | 11/8/17 |
| M22 | 20.5 | NA | TFSCS026 | Thai | FALSE | 6 | TD.86489 | LowFiber | 14.6 | 208 | 11/8/17 |
| M23 | 21.2 | NA | TFSCS026 | Thai | FALSE | 6 | TD.86489 | LowFiber | 14.6 | 208 | 11/8/17 |
| M24 | 20.8 | NA | TFSCS026 | Thai | FALSE | 6 | TD.86489 | LowFiber | 14.6 | 208 | 11/8/17 |
| M27 | 18.9 | NA | IMP.264 | US | FALSE | 7 | TD.86489 | LowFiber | 7.8 | 209.6 | 11/8/17 |
| M28 | 19.4 | NA | IMP.264 | US | FALSE | 7 | TD.86489 | LowFiber | 7.8 | 209.6 | 11/8/17 |
| M29 | 22.3 | NA | IMP.264 | US | FALSE | 7 | TD.86489 | LowFiber | 7.8 | 209.6 | 11/8/17 |
| M30 | 23.5 | NA | IMP.264 | US | FALSE | 7 | TD.86489 | LowFiber | 7.8 | 209.6 | 11/8/17 |
| M21 | 20.6 | NA | TFSCS026 | Thai | FALSE | 6 | TD.86489 | LowFiber | 58.3 | 181.2 | 11/22/17 |
| M22 | 20.9 | NA | TFSCS026 | Thai | FALSE | 6 | TD.86489 | LowFiber | 58.3 | 181.2 | 11/22/17 |
| M23 | 22 | NA | TFSCS026 | Thai | FALSE | 6 | TD.86489 | LowFiber | 58.3 | 181.2 | 11/22/17 |
| M24 | 21.9 | NA | TFSCS026 | Thai | FALSE | 6 | TD.86489 | LowFiber | 58.3 | 181.2 | 11/22/17 |
| M27 | 20.4 | NA | IMP.264 | US | FALSE | 7 | TD.86489 | LowFiber | 76.3 | 198.6 | 11/22/17 |
| M29 | 20.7 | NA | IMP.264 | US | FALSE | 7 | TD.86489 | LowFiber | 76.3 | 198.6 | 11/22/17 |
| M30 | 23.8 | NA | IMP.264 | US | FALSE | 7 | TD.86489 | LowFiber | 76.3 | 198.6 | 11/22/17 |
| M21 | 21.2 | NA | TFSCS026 | Thai | FALSE | 6 | TD.86489 | LowFiber | 7.6 | 255.5 | 12/6/17 |
| M22 | 21.5 | NA | TFSCS026 | Thai | FALSE | 6 | TD.86489 | LowFiber | 7.6 | 255.5 | 12/6/17 |
| M23 | 22.3 | NA | TFSCS026 | Thai | FALSE | 6 | TD.86489 | LowFiber | 7.6 | 255.5 | 12/6/17 |
| M24 | 22.4 | NA | TFSCS026 | Thai | FALSE | 6 | TD.86489 | LowFiber | 7.6 | 255.5 | 12/6/17 |
| M27 | 20.1 | NA | IMP.264 | US | FALSE | 7 | TD.86489 | LowFiber | 66.6 | 197.2 | 12/6/17 |
| M29 | 21.9 | NA | IMP.264 | US | FALSE | 7 | TD.86489 | LowFiber | 66.6 | 197.2 | 12/6/17 |
| M30 | 24 | NA | IMP.264 | US | FALSE | 7 | TD.86489 | LowFiber | 66.6 | 197.2 | 12/6/17 |
| M21 | 22.4 | 173 | TFSCS026 | Thai | FALSE | 6 | TD.86489 | LowFiber | 83.8 | NA | 12/20/17 |
| M22 | 22 | 172 | TFSCS026 | Thai | FALSE | 6 | TD.86489 | LowFiber | 83.8 | NA | 12/20/17 |
| M23 | 23.7 | 182 | TFSCS026 | Thai | FALSE | 6 | TD.86489 | LowFiber | 83.8 | NA | 12/20/17 |
| M24 | 23.6 | 98 | TFSCS026 | Thai | FALSE | 6 | TD.86489 | LowFiber | 83.8 | NA | 12/20/17 |
| M27 | 21 | 249 | IMP.264 | US | FALSE | 7 | TD.86489 | LowFiber | 67.5 | NA | 12/20/17 |
| M29 | 23 | 228 | IMP.264 | US | FALSE | 7 | TD.86489 | LowFiber | 67.5 | NA | 12/20/17 |
| M30 | 24.8 | 155 | IMP.264 | US | FALSE | 7 | TD.86489 | LowFiber | 67.5 | NA | 12/20/17 |
| M31 | 19 | 187 | IMP.264 | US | FALSE | 8 | LabDiet 5061 | HighFiber | NA | 503.6 | 1/16/18 |
| M32 | 16.5 | 163 | IMP.264 | US | FALSE | 8 | LabDiet 5061 | HighFiber | NA | 503.6 | 1/16/18 |
| M33 | 16.8 | 131 | IMP.264 | US | FALSE | 8 | LabDiet 5061 | HighFiber | NA | 503.6 | 1/16/18 |
| M34 | 17.8 | 146 | IMP.264 | US | FALSE | 8 | LabDiet 5061 | HighFiber | NA | 503.6 | 1/16/18 |
| M31 | 21.4 | NA | IMP.264 | US | FALSE | 8 | LabDiet 5061 | HighFiber | 262 | 323.1 | 1/30/18 |
| M32 | 18.7 | NA | IMP.264 | US | FALSE | 8 | LabDiet 5061 | HighFiber | 262 | 323.1 | 1/30/18 |
| M33 | 20.1 | NA | IMP.264 | US | FALSE | 8 | LabDiet 5061 | HighFiber | 262 | 323.1 | 1/30/18 |
| M34 | 19 | NA | IMP.264 | US | FALSE | 8 | LabDiet 5061 | HighFiber | 262 | 323.1 | 1/30/18 |
| M31 | 22.2 | NA | IMP.264 | US | FALSE | 8 | LabDiet 5061 | HighFiber | 110.3 | 250.8 | 2/13/18 |
| M32 | 19.9 | NA | IMP.264 | US | FALSE | 8 | LabDiet 5061 | HighFiber | 110.3 | 207.3 | 2/13/18 |
| M33 | 20.6 | NA | IMP.264 | US | FALSE | 8 | LabDiet 5061 | HighFiber | 110.3 | 207.3 | 2/13/18 |
| M34 | 22.1 | NA | IMP.264 | US | FALSE | 8 | LabDiet 5061 | HighFiber | 110.3 | 250.8 | 2/13/18 |
| M31 | 23.8 | NA | IMP.264 | US | FALSE | 9 | LabDiet 5061 | HighFiber | 128.1 | 231.5 | 2/27/18 |
| M32 | 21.9 | NA | IMP.264 | US | FALSE | 8 | LabDiet 5061 | HighFiber | 89.4 | 232.9 | 2/27/18 |
| M33 | 23.2 | NA | IMP.264 | US | FALSE | 8 | LabDiet 5061 | HighFiber | 89.4 | 232.9 | 2/27/18 |
| M34 | 21.5 | NA | IMP.264 | US | FALSE | 9 | LabDiet 5061 | HighFiber | 128.1 | 231.5 | 2/27/18 |
| M31 | 23.3 | 204 | IMP.264 | US | FALSE | 9 | LabDiet 5061 | HighFiber | 110.2 | NA | 3/13/18 |
| M32 | 22.3 | 212 | IMP.264 | US | FALSE | 8 | LabDiet 5061 | HighFiber | 114.8 | NA | 3/13/18 |
| M33 | 21.1 | 254 | IMP.264 | US | FALSE | 8 | LabDiet 5061 | HighFiber | 114.8 | NA | 3/13/18 |
| M34 | 21.4 | 171 | IMP.264 | US | FALSE | 9 | LabDiet 5061 | HighFiber | 110.2 | NA | 3/13/18 |
| M35 | 19.2 | 138 | TFSCS026 | Thai | FALSE | 10 | LabDiet 5061 | HighFiber | NA | 223.9 | 2/13/18 |
| M36 | 19.7 | 133 | TFSCS026 | Thai | FALSE | 10 | LabDiet 5061 | HighFiber | NA | 223.9 | 2/13/18 |
| M37 | 17.4 | 122 | TFSCS026 | Thai | FALSE | 10 | LabDiet 5061 | HighFiber | NA | 223.9 | 2/13/18 |
| M38 | 18.9 | 153 | TFSCS026 | Thai | FALSE | 10 | LabDiet 5061 | HighFiber | NA | 223.9 | 2/13/18 |
| M35 | 19.4 | NA | TFSCS026 | Thai | FALSE | 10 | LabDiet 5061 | HighFiber | 101.4 | 431.4 | 2/27/18 |
| M36 | 19.9 | NA | TFSCS026 | Thai | FALSE | 10 | LabDiet 5061 | HighFiber | 101.4 | 431.4 | 2/27/18 |
| M37 | 18 | NA | TFSCS026 | Thai | FALSE | 10 | LabDiet 5061 | HighFiber | 101.4 | 431.4 | 2/27/18 |
| M38 | 19.6 | NA | TFSCS026 | Thai | FALSE | 10 | LabDiet 5061 | HighFiber | 101.4 | 431.4 | 2/27/18 |
| M35 | 20.8 | NA | TFSCS026 | Thai | FALSE | 10 | LabDiet 5061 | HighFiber | 90.2 | 313 | 3/13/18 |
| M36 | 21 | NA | TFSCS026 | Thai | FALSE | 10 | LabDiet 5061 | HighFiber | 90.2 | 313 | 3/13/18 |
| M37 | 19.2 | NA | TFSCS026 | Thai | FALSE | 10 | LabDiet 5061 | HighFiber | 90.2 | 313 | 3/13/18 |
| M38 | 20.7 | NA | TFSCS026 | Thai | FALSE | 10 | LabDiet 5061 | HighFiber | 90.2 | 313 | 3/13/18 |
| M35 | 22.4 | NA | TFSCS026 | Thai | FALSE | 10 | LabDiet 5061 | HighFiber | 63 | 356 | 3/27/18 |
| M36 | 21.7 | NA | TFSCS026 | Thai | FALSE | 10 | LabDiet 5061 | HighFiber | 63 | 356 | 3/27/18 |
| M37 | 21 | NA | TFSCS026 | Thai | FALSE | 10 | LabDiet 5061 | HighFiber | 63 | 356 | 3/27/18 |
| M38 | 21.7 | NA | TFSCS026 | Thai | FALSE | 10 | LabDiet 5061 | HighFiber | 63 | 356 | 3/27/18 |
| M35 | 23.4 | 187 | TFSCS026 | Thai | FALSE | 10 | LabDiet 5061 | HighFiber | 111.9 | NA | 4/10/18 |
| M36 | 22.3 | 160 | TFSCS026 | Thai | FALSE | 10 | LabDiet 5061 | HighFiber | 111.9 | NA | 4/10/18 |
| M37 | 20.8 | 231 | TFSCS026 | Thai | FALSE | 10 | LabDiet 5061 | HighFiber | 111.9 | NA | 4/10/18 |
| M38 | 22.8 | 114 | TFSCS026 | Thai | FALSE | 10 | LabDiet 5061 | HighFiber | 111.9 | NA | 4/10/18 |
