# Supplementary material for: Industrialized human gut microbiota increases CD8+ T cells and mucus thickness in humanized mouse gut
Source: Gut Microbes. 2023 Oct 18;15(2):2266627. doi: 10.1080/19490976.2023.2266627 (PMC10588527; doi:10.1080/19490976.2023.2266627)
Supplement: Supplemental Material [file KGMI_A_2266627_SM0757.zip › Supplemental tables and figures/Supplemental Figures.docx]

Cell count normalized to

Supplemental Figure 1


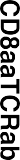

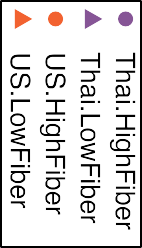

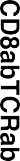
CD45+ counts


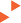

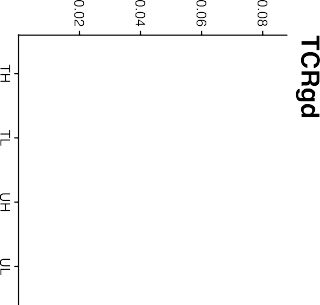

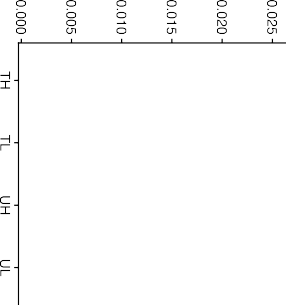

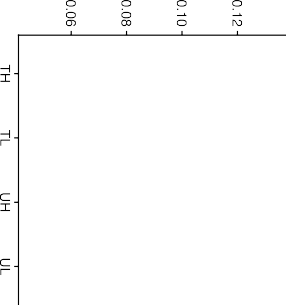

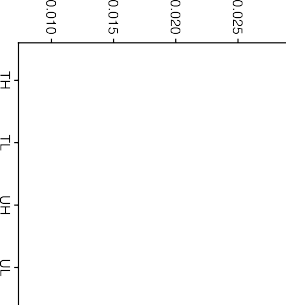


# A

0.8

0.6

0.4

0.2

0.0

0.75

0.50

0.25

0.00

# B

**M21**

2 4 6 8

**M35**

2 4 6 8

0.75

0.50

0.25

0.00

1.00

0.75

0.50

0.25

0.00

**M22**

2 4 6 8

**M36**

2 4 6 8

Week

0.8

0.6

0.4

0.2

0.0

1.00

0.75

0.50

0.25

0.00

**M23**

2 4 6 8

**M37**

2 4 6 8

0.75

0.50

0.25

0.00

0.75

0.50

0.25

0.00

Akkermansia muciniphila Bacteroides

Bacteroides fragilis Bacteroides dorei Romboutsia timonensis Lactobacillus Bacteroides uniformis Parabacteroides gordonii

**M24**

2

4 6

8

**M38**

2

4

6

8

Parabacteroides distasonis Lachnoclostridium [Clostridium] innocuum Subdoligranulum

Parasutterella excrementihominis Bacteroides caccae

Alistipes putredinis Enterobacteriaceae Enterococcus Clostridium Sutterella Lactococcus Ruminococcus Bacteroides stercoris Parabacteroides

Paraclostridium bifermentans Morganellaceae

Morganella morganii Clostridium butyricum Paeniclostridium sordellii Streptococcus

[Clostridium] glycyrrhizinilyticum Clostridium perfringens Bacteroides nordii

Bacteroides xylanisolvens Bacteroides ovatus Clostridium colicanis Odoribacter splanchnicus [Clostridium] aldenense Prevotella

0.125

Mucus Thickness

0.100

0.075

0.050

0.1 0.2 0.3 0.4 0.5

Akkermansia cor=−0.2 P=0.3

0.25

0.20

0.15

0.10

0.05

#### Subdoligranulum

0.2

0.1

#### Romboutsia

0.08

0.06

0.04

0.02

#### Butyricicoccus

0.4

0.3

0.2

0.1

#### Lactococcus

0.25

0.20

0.15

0.10

0.05

0.00

0.10

0.05

TH TL UH UL


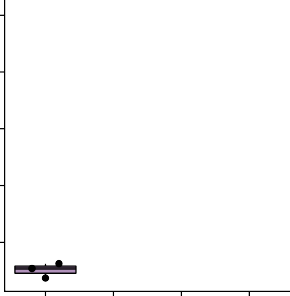

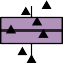

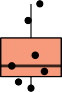

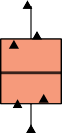


#### Gemmiger


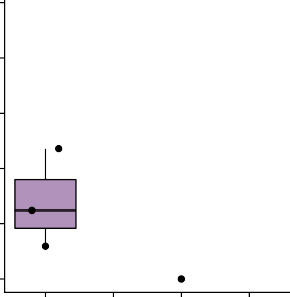

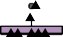

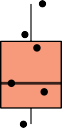

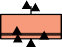


TH TL UH UL

#### Flavonifractor


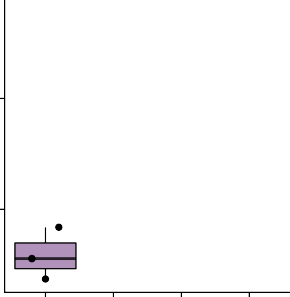

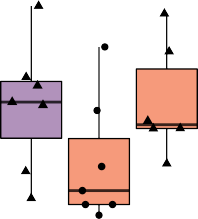


0.0

0.3


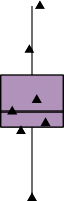

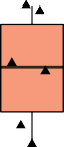

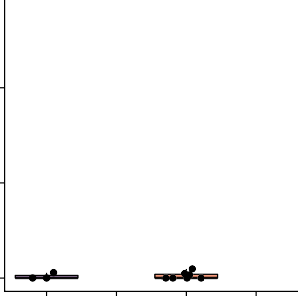

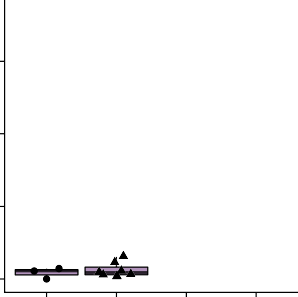

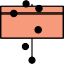

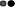

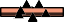


0.2

0.1

0.0

0.30

0.25

0.20

0.15

0.10

TH TL UH UL

#### Roseburia

TH TL UH UL

####
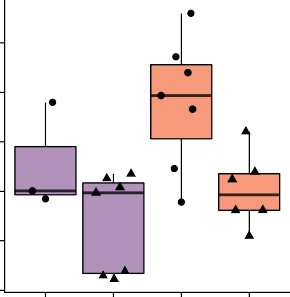
Blautia

0.100

0.075

0.050

0.025

0.000

0.015

0.010

0.005


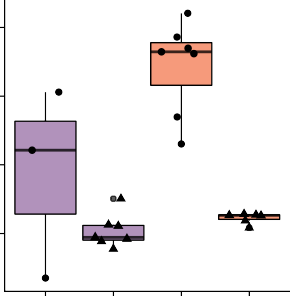
TH TL UH UL

#### Dorea


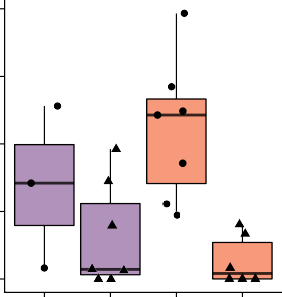


TH TL UH UL

####
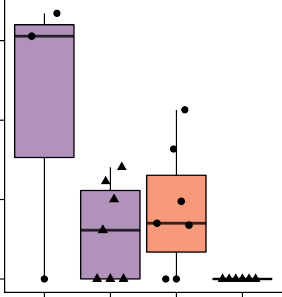
Anaerosporobacter

0.0

0.15


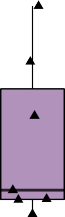

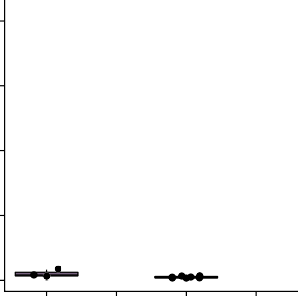

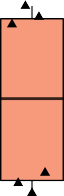


0.10

0.05

0.00

0.10

0.05

TH TL UH UL

#### Enterococcus


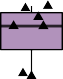

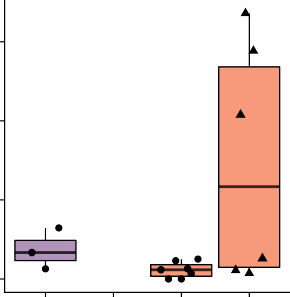


TH TL UH UL

#### Terrisporobacter

0.04

0.03

0.02

0.01

TH TL UH UL

#### Ihubacter


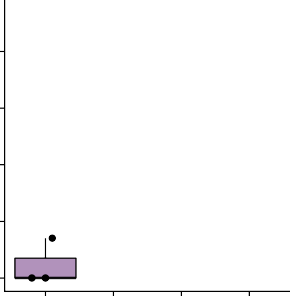

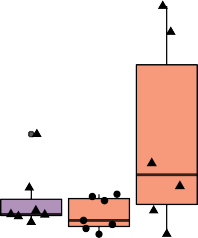


0.05

0.08

0.06

0.04

0.02

TH TL UH UL

#### Succinatimonas

0.000

0.04


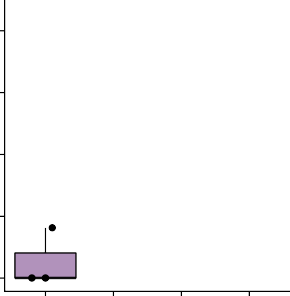

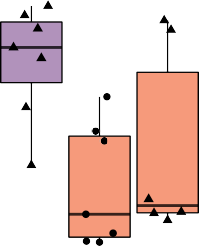


0.03

0.02

0.01

TH TL UH UL

#### Eggerthella

0.00


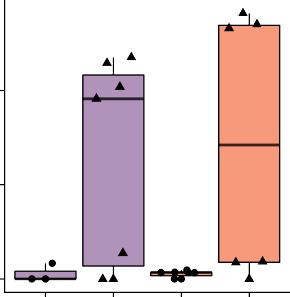
0.100

0.075

0.050

0.025

TH TL UH UL

#### Muricomes


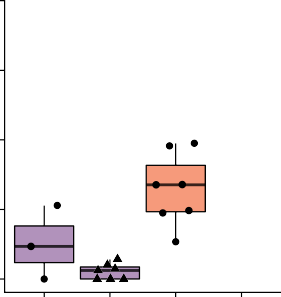

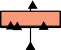

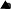


0.00

0.08

0.06

0.04

0.02

0.00

TH TL UH UL

#### Negativibacillus


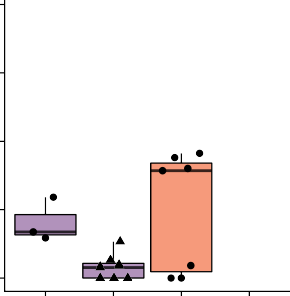

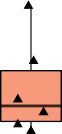


TH TL UH UL

0.00


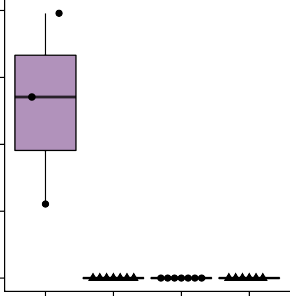
0.100

0.075

0.050

0.025

TH TL UH UL

#### Collinsella


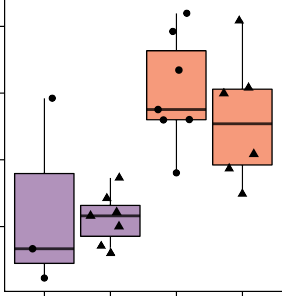


TH TL UH UL

0.00

TH TL UH UL


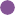

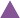

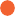

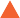


Thai.HighFiber Thai.LowFiber US.HighFiber US.LowFiber

0.000

TH TL UH UL

# A

## Donor Pair 1


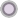


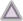

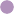
0.25


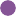

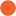

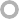


Thai.HighFiber Thai.LowFiber US.HighFiber US.LowFiber CH-HighFiber

CH-LowFiber

PC2 [15.23%]


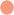
0.00


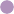

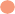

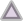

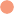


−0.25


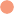


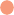


# B

1.00

RelativeAbundance

0.75

0.50

0.25

0.00

Thai.LowFiber

CH−LowFiber

−0.5 0.0 0.5

PC1 [60.12%]

## Donor Pair 1

Desulfovibrio Roseburia Romboutsia Tyzzerella Subdoligranulum Erysipelatoclostridium Paraprevotella Hungatella Lachnoclostridium Blautia Faecalibacterium Lactococcus Parabacteroides Akkermansia Bacteroides

Thai.HighFiber

US.HighFiber

CH−HighFiber

US.LowFiber

**A**


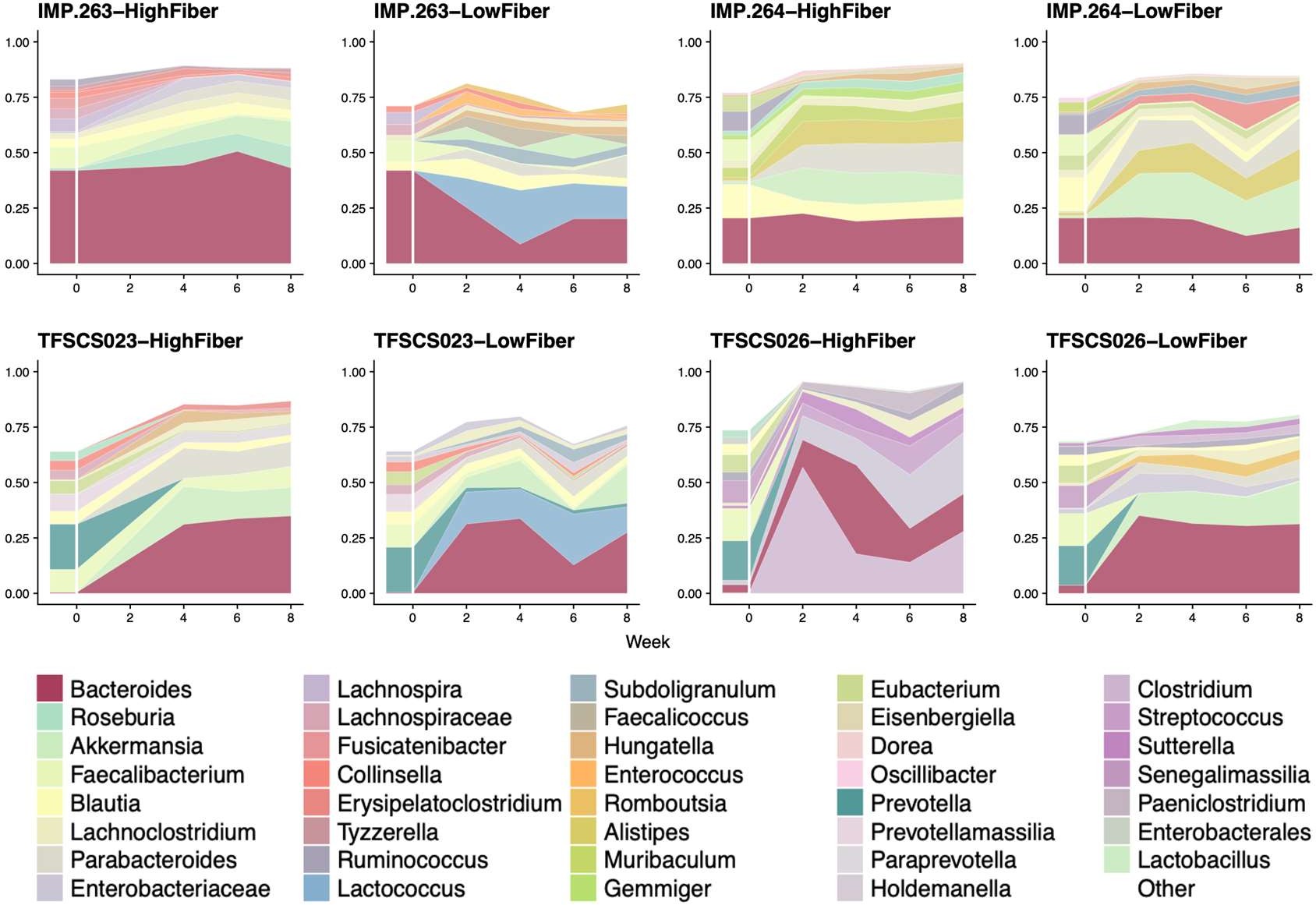


**USDonor1-HF**

**USDonor1-LF**

**USDonor2-HF**

**USDonor2-LF**

**ThaiDonor2-LF**

**ThaiDonor2-HF**

**ThaiDonor1-LF**

**ThaiDonor1-HF**

**B**


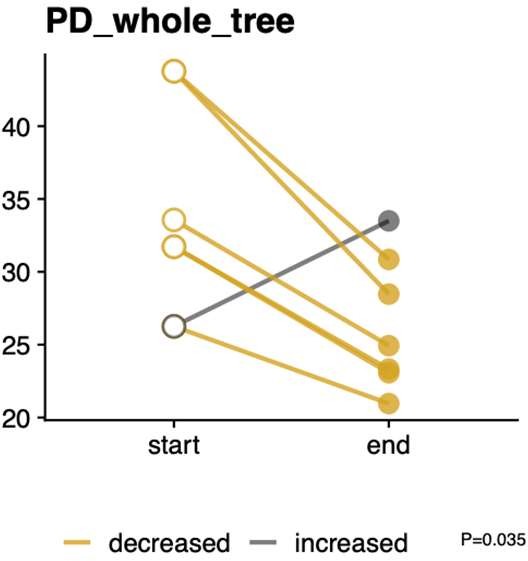


**Faith's phylogenetic diversity**

Donor Sample group

samples mean

**A Beta diversity at first and last B**


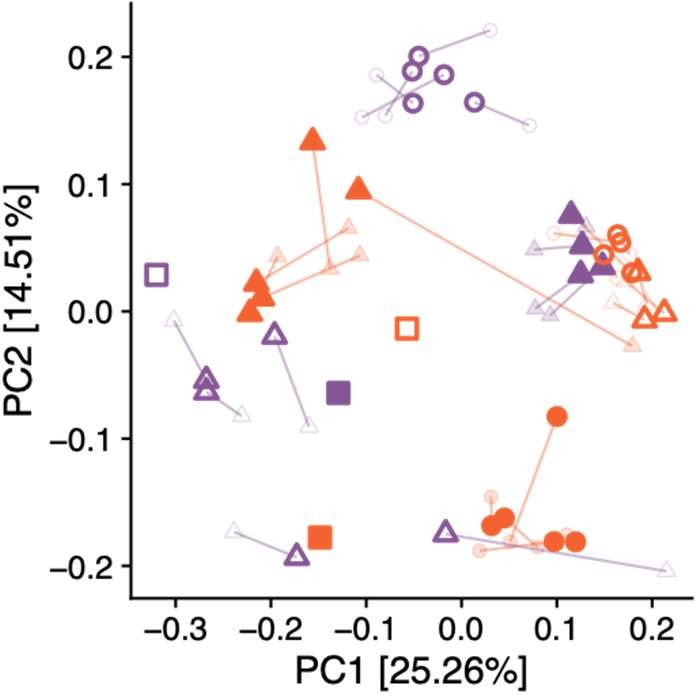
**timepoints (Unweighted UniFrac)**


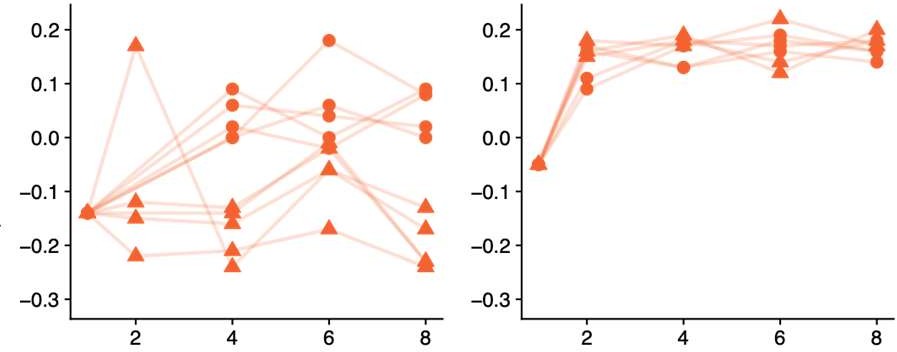
**US Donor 1 US Donor 2**


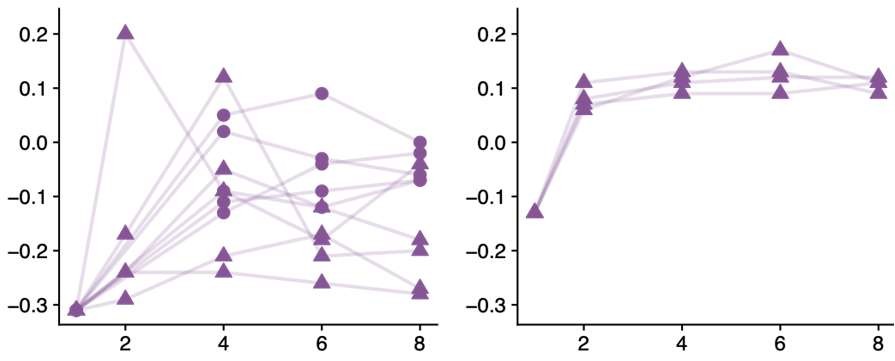
**Thai Donor 1 Thai Donor 2**

PC1 (unweighted UniFrac)

###
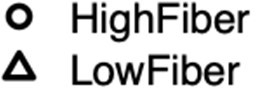

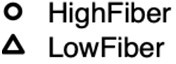
Week


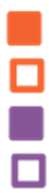


**US Donor 1**

**US Donor 2**

**Thai Donor 1**

**Thai Donor 2**


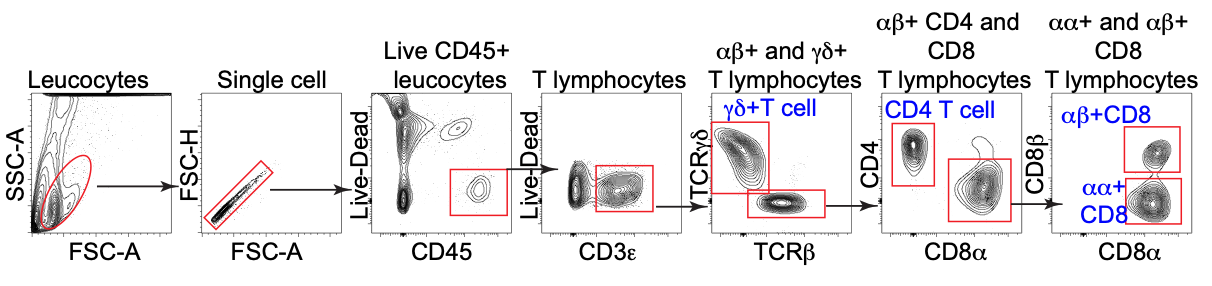


Supplemental Figure 7
