# Supplementary material for: Industrialized human gut microbiota increases CD8+ T cells and mucus thickness in humanized mouse gut
Source: Gut Microbes. 2023 Oct 18;15(2):2266627. doi: 10.1080/19490976.2023.2266627 (PMC10588527; doi:10.1080/19490976.2023.2266627)
Supplement: Supplemental Material [file KGMI_A_2266627_SM0757.zip › Supplemental tables and figures/Supplemental tables and figures captions.docx]

# Supplemental Figure Legends

## Figure S1. Immune responses are dependent on donor microbiota in the small intestine lamina propria

Points represent counts of live immune cell types normalized by total count of live CD45+ cells per mouse small intestine (week 8, n = 4 mice were excluded in the NH group). TCRγδ and CD8ααTCRαβ+ T cells are significantly elevated in US-donor groups (two-way ANOVA, donor P = 0.024 and P = 0.015, respectively).

## Figure S2. Taxonomic composition of infected mice

 (S2A) Taxonomic plots of mouse pellets collected over 8 weeks from mice gavaged with material from donor TFSCS026 and fed a low-fiber diet (top row, M21-M24) or high-fiber diet (bottom row, M35-M38, infected mice).

(S2B) Mucus thickness measurements show a negative correlation trend that is not significantly associated with the relative abundance of *Akkermansia* (P=0.30, Pearson’s correlation coefficient = -0.20).

## Figure S3. Diet is a primary driver of differential taxa across sample groups.

Relative abundances of taxa that have been identified as differentially significant based on a two-way ANOVA test (diet or donor type variable with FDR-corrected q-value < 0.10) are shown. q-values for all taxa can be found in Supplemental Table 1. Cohoused and n=4 infected mice samples are excluded.

## Figure S4. Effects of cohousing on microbiota differ by diet

(S4A) PCoA of mouse microbiota (weighted Unifrac) from donor Pair 1 show diet-related changes in microbiota composition after cohousing. Comparisons of cohoused groups to their respective original groups resulted in no differences in taxonomic composition between TH and CH, suggesting that cohousing on a high fiber diet supports a Thai-donor microbiota (whereas 43 significantly differential taxa were found between UH and CH groups). Cohoused groups that continued on a low fiber diet became more similar to the UL group, with 11 more taxa shared between UL and CL (24 taxa were significantly different between TL and CL, and 13 taxa were significantly different between UL and CL). Comparisons were performed using a two-sample *t-test* on asin-sqrt relative abundance genus-level taxa tables (taxa with less than 10% prevalence across samples were removed), corrected for multiple comparisons using FDR, and noted as significantly different if q < 0.10. Samples were collected at study endpoints (week 8 or 10, depending on whether or not the mouse was cohoused). TH=Thailand-Donor-High-Fiber; TL=Thailand-Donor-Low-Fiber; UH=US-donor-High-Fiber; UL=US-Donor-Low-Fiber; CH=High-Fiber-Cohoused; CL=Low-Fiber-Cohoused.

(S4B) The relative abundances of the 15 most prevalent taxa within donor pair 1 are shown. Samples were collected at study endpoints (week 8 or 10, depending on whether or not the mouse was cohoused). TH=Thailand-Donor-High-Fiber; UH=US-Donor-High-Fiber; CH=Cohoused TH and UH; TL=Thailand-Donor-Low-Fiber; UL=US-Donor-Low-Fiber; CL=Cohoused TL and UL.

## Figure S5. Donor microbiome composition and alpha diversity are partially preserved in sample groups immediately post-colonization.

(S5A) Taxa summary plots calculated at the genus level are shown over time and grouped by individual donor and diet. Donor microbiomes are shown at Week 0 (before the vertical line) for comparison with donor material and the subsequent microbiome samples. The top 15 most prevalent taxa across timepoints within each group are shown per plot, with all other taxa collapsed as “Other” and omitted for visualization purposes. The genera and colors shown in the legend are shared across all plots. Note that the ThaiDonor2-HF group contains the infected mice. Samples from cohoused mice have been excluded from this analysis.

(S5B) Alpha diversity (Faith’s phylogenetic diversity) of the donor microbiome and mean alpha diversity of the first available samples post-gavage per donor-diet group are shown. Decreases in diversity are highlighted in yellow, and increases are highlighted in black. The relative alpha diversity of the donors is maintained in the sample groups, with the exception of the US Donor 1 - Low Fiber diet group, which exhibited a large increase in alpha diversity post-gavage. Samples from cohoused and infected mice have been excluded from this analysis.

## Figure S6. Longitudinal microbiome dynamics vary across sample groups.

(S6A) PCoA plot showing unweighted UniFrac distances between donor microbiomes (square points), first available microbiomes (transparent points), and last available microbiomes (solid or outlined circles and triangles). Points are colored by donor type and shapes represent diets. The first available microbiomes (sampled at week 2 or 4) and last available microbiomes (sampled at week 8) for each mouse is connected with a line. Samples from cohoused and infected mice have been excluded from this analysis.

(S6B) The first principal coordinate of the PCoA (PC1) generated in Figure S6A is plotted over time. Plots are separated by individual donors, with every line starting with the respective donor sample (at time 0). Weeks 2-8 show the changes in the individual mouse microbiomes according to PC1. Samples from cohoused and infected mice have been excluded from this analysis.

## Figure S7. Gating strategy for T cell populations.

(S7) A representative gating strategy to identify various T lymphocyte populations within the intestinal epithelium is shown. Cells within the red rectangles of each flow plot are listed above each plot and the four separate cell types that have been enumerated in this study are listed in blue within each plot.

# Supplemental Table Legends

## Table S1. Summary table of bi-weekly mice measurements

Fasting blood glucose, mouse body weight, and food weight measurements taken throughout the duration of this study. On 11/2/17, 11/6/17, and 11/22/17, three mice (M26, M25, M28, respectively) experienced unexplained deaths and were removed from their cages with sterile gloves. Note that these events are unrelated to the infected mice M35-M38.

## Table S2. Differential taxa across sample groups.

A two-way ANOVA test with diet type and donor type as variables (and controlling for donor sample) was performed on normalized (asin-sqrt) relative abundance genus-level taxa tables (taxa with less than 10% prevalence across samples were removed). q-values shown represent p-values that have been adjusted for multiple comparisons by FDR. Cohoused and n=4 infected mice samples are excluded. Values have been sorted by donor and diet q-values.
